# Constructing exact representations of quantum many-body systems with deep neural networks

**DOI:** 10.1038/s41467-018-07520-3

**Published:** 2018-12-14

**Authors:** Giuseppe Carleo, Yusuke Nomura, Masatoshi Imada

**Affiliations:** 1 Center for Computational Quantum Physics, Flatiron Institute, 162 5th Avenue, New York, NY 10010 USA; 2 Institute for Theoretical Physics, ETH Zurich, Wolfgang-Pauli-Str. 27, 8093 Zurich, Switzerland; 3 Department of Applied Physics, The University of Tokyo, 7-3-1 Hongo, Bunkyo-ku, Tokyo 113-8656 Japan

## Abstract

Obtaining accurate properties of many-body interacting quantum matter is a long-standing challenge in theoretical physics and chemistry, rooting into the complexity of the many-body wave-function. Classical representations of many-body states constitute a key tool for both analytical and numerical approaches to interacting quantum problems. Here, we introduce a technique to construct classical representations of many-body quantum systems based on artificial neural networks. Our constructions are based on the deep Boltzmann machine architecture, in which two layers of hidden neurons mediate quantum correlations. The approach reproduces the exact imaginary-time evolution for many-body lattice Hamiltonians, is completely deterministic, and yields networks with a polynomially-scaling number of neurons. We provide examples where physical properties of spin Hamiltonians can be efficiently obtained. Also, we show how systematic improvements upon existing restricted Boltzmann machines *ansatze* can be obtained. Our method is an alternative to the standard path integral and opens new routes in representing quantum many-body states.

## Abstract (web summary)

Significant improvements in numerical methods for quantum systems often come from finding new ways of representing quantum states that can be optimized and simulated more efficiently. Here the authors demonstrate a method to calculate exact neural network representations of many-body ground states.

## Introduction

A tremendous amount of successful developments in quantum physics builds upon the mapping between many-body quantum systems and effective classical theories. The probably most well-known mapping is due to Feynman, who introduced an exact representation of many-body quantum systems in terms of statistical summations over classical particles trajectories^1^. Effective classical representations of quantum many-body systems are however not unique, and other approaches rely on different inspiring principles, such as perturbative expansions^2^, or decomposition of interactions with auxiliary degrees of freedom^3,4^. The classical representations of quantum states allow both for novel conceptual developments and efficient numerical simulations. On one hand, perturbative approaches based on the graphical resummation of classes of diagrams are at the heart of many-body analytical approaches in various fields of research, ranging from particle to condensed-matter physics^5^. On the other hand, several non-perturbative numerical methods for many-body quantum systems are also based on these mappings. Quantum Monte Carlo (QMC) methods are among the most successful numerical techniques, relying on continuous-space polymer representations^6–9^, world-line lattice path integrals^10,11^, continuous time algorithm^12^, and summation of perturbative diagrams^13,14^. Effective classical representations are also the building block of variational methods based on correlated many-body wave-functions^15^. Several successful variational techniques make extensive use of parametric representations of quantum states, where the effective parameters are determined by means of the variational principle^16–19^. In matrix-product and tensor-network-states the ground-state is expressed as a classical network^20,21^. In general, finding alternative, efficient classical representations of quantum states can help establishing novel numerical and analytical techniques to study challenging open issues.

Recently, an efficient variational representation of many-body systems in terms of artificial neural networks, which consists of classical degrees of freedom, has been introduced^22^. Numerical results have shown that artificial neural networks can represent many-body states with high accuracy^22–31^. The majority of the variational approaches adopted so-far are based on shallow neural networks, called restricted Boltzmann machines (RBM), in which the physical degrees of freedom interact with an ensemble of hidden degrees of freedom (neurons). While shallow RBM states have promising features in terms of entanglement capacity^25,32–34^, only deep networks are guaranteed to provide a complete and efficient description of the most general quantum states^35,36^.

In this work, we introduce a constructive approach to explicitly generate deep network structures corresponding to exact quantum many-body ground states. We demonstrate this construction for interacting lattice spin models, including the transverse-field Ising and Heisenberg models. Our constructions are fully deterministic, in stark contrast to the shallow RBM case, in which the numerical optimization of the network parameters is inevitable. The number of neurons required in the construction scales only polynomially with the system size, thus the present approach constitutes a new family of efficient quantum-to-classical mappings exhibiting a prominent representational flexibility. Given as a simple set of iterative rules, these constructions can be used both as a self-standing tool, or to systematically improve results obtained with variational shallow networks. The latter improves the efficiency of the method because the numerically optimized shallow RBM states are already good approximations for ground states. Finally, we discuss sampling strategies from the generated deep networks and show numerical results for one-dimensional spin models.

## Results

### General scheme of constructing deep neural states

The ground state of a generic Hamiltonian, ${\cal H}$, can be found through imaginary-time evolution, $\left| {{\mathrm{\Psi }}(\tau )} \right\rangle$ = ${\mathrm {e}}^{ - \tau {\cal H}}\left| {{\mathrm{\Psi }}_0} \right\rangle$, for a sufficiently large $\tau \gg {\mathrm{\Delta }}E^{ - 1}$. Here Δ*E* is the energy gap between the ground and the first excited state, $\left| {{\mathrm{\Psi }}_0} \right\rangle$ is an arbitrary initial state non-orthogonal to the exact ground state, and we work in units where *ħ* = 1. For a finite system, the energy gap is typically finite, and the total propagation time needed to reach the ground state within an arbitrary given accuracy is expected to grow at most polynomially with the system size (for systems becoming gapless in the thermodynamic limit).

**Fig. 1:**
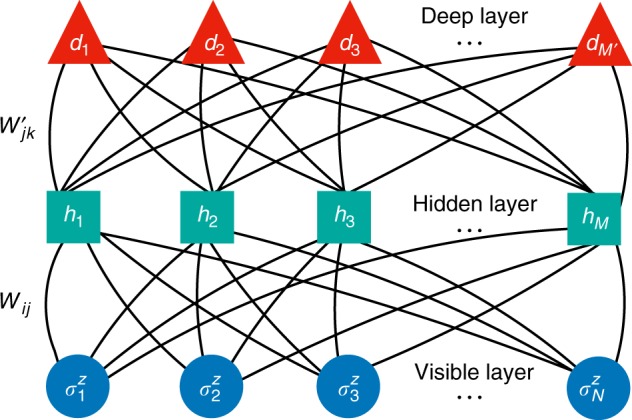
Structure of deep Boltzmann machine. Dots, squares, and triangles represent physical degrees of freedom $\left( {\sigma _i^z} \right)$, hidden units (*h*_*j*_), deep units (*d*_*k*_), respectively. Solid curves represent interlayer couplings (*W*_*ij*_ and $W_{jk}^\prime$)

Here, we introduce a representation of the wave-function coefficients in terms of a deep Boltzmann machine (DBM)^37^. For the sake of concreteness, let us consider the case of *N* spins, described by the quantum numbers $\left| {\sigma ^z} \right\rangle$ = $\left| {\sigma _1^z \ldots \sigma _N^z} \right\rangle$. Then, we represent generic many-body amplitudes $\left\langle {\sigma _1^z \ldots \sigma _N^z{\mathrm{|\Psi }}} \right\rangle \equiv {\mathrm{\Psi }}\left( {\sigma ^z} \right)$ in the two-layer DBM form:

1 $$\begin{array}{c}{\mathrm{\Psi }}_{\cal W}\left( {\sigma ^z} \right) = \mathop {\sum}\limits_{\{ h,d\} } {\kern 1pt} {\mathrm{exp}}\left[ {\mathop {\sum}\limits_i {\kern 1pt} a_i\sigma _i^z + \mathop {\sum}\limits_{ij} {\kern 1pt} \sigma _i^zW_{ij}h_j} \right.\\ \left. { + \mathop {\sum}\limits_j {\kern 1pt} b_jh_j + \mathop {\sum}\limits_{jk} {\kern 1pt} h_jd_kW_{jk}^\prime + \mathop {\sum}\limits_k {\kern 1pt} b_k^\prime d_k} \right]\end{array}$$

where we have introduced *M* hidden units *h*, *M*′ deep units *d*, and a set of couplings and bias terms ${\cal W}$ ≡ (*a*, *b*, *b*′, *W*, *W*′). A sketch of the DBM architecture is shown in Fig. 1.

In the following, we specialize to the case of spin 1/2, thus all the units are taken to be *σ*^*z*^, *h*, *d* = ±1. This representation is the natural deep-network generalization of the shallow RBM, introduced as variational ansatz in ref. ^22^. As for the RBM form, also in this case direct connections between variables in the same layer are not allowed. A crucial difference is however that the layer of deep variables makes, in general, the evaluation of the wave-function amplitudes not possible analytically. At variance with RBM, the DBM form is known to be universal, as proven by Gao and Duan recently^35^.

Our key finding is that, thanks to the much more flexible representability, the DBM wave function can reproduce the Hamiltonian imaginary-time evolution exactly by changing its form dynamically, and that the parameters for ground state DBM network can be derived analytically. In order to find explicit expressions for the parameters ${\cal W}$ that represent $\left| {{\mathrm{\Psi }}(\tau )} \right\rangle$ for arbitrary imaginary time, we start considering a second-order Trotter–Suzuki decomposition^10,38^:

2 $$\left| {{\mathrm{\Psi }}(\tau )} \right\rangle = {\cal G}_1\left( {\delta _\tau {\mathrm{/}}2} \right){\cal G}_2\left( {\delta _\tau } \right) \ldots {\cal G}_1\left( {\delta _\tau } \right){\cal G}_2\left( {\delta _\tau } \right){\cal G}_1\left( {\delta _\tau {\mathrm{/}}2} \right)\left| {{\mathrm{\Psi }}_0} \right\rangle ,$$

where we have decomposed the Hamiltonian into two non-commuting parts, ${\cal H} = {\cal H}_1 + {\cal H}_2$, and introduced the short-time propagators ${\cal G}_\nu \left( {\delta _\tau } \right) = {\mathrm {e}}^{ - {\cal H}_\nu \delta _\tau }$. The problem of finding an exact representation for $\left| {{\mathrm{\Psi }}(\tau )} \right\rangle$ then reduces to finding a rule to construct the building blocks of the time-evolution, namely representing the state after two-types of propagators by DBM with new parameters $\bar {\cal W}$:

3 $${\mathrm {e}}^{ - {\cal H}_\nu \delta _\tau }\left| {\Psi _{\cal W}} \right\rangle = C\left| {{\mathrm{\Psi }}_{\bar {\cal W}}} \right\rangle .$$

In practice, this is achieved either by changing parameters ${\cal W}$, or by introducing additional parameters in ${\cal W}$, adding new neurons and creating new connections in the network.

In the following, we show concrete examples for paradigmatic spin hamiltonians, namely the transverse-field Ising and Heisenberg models. The rest of this section provides a general overview of how the DBM constructions are derived (how Eq. (3) is satisfied) for these models. The next section (Sampling strategies) discusses how they can be used in numerical schemes. A complete, in-depth derivation of the representations and algorithms can be found both in Methods and in the Supplementary Notes, as referred to at each step in this section. Furthermore, we provide computer codes to create the DBM network for each model as Supplementary Software 1–4.

### Transverse-field Ising model (TFIM)

We start considering the TFIM on an arbitrary interaction graph. In this case, we decompose the Hamiltonian into two parts: ${\cal H}_1 = - \mathop {\sum}\nolimits_l {\kern 1pt} {\mathrm{\Gamma }}_l\sigma _l^x$, and ${\cal H}_2 = \mathop {\sum}\nolimits_{l < m} {\kern 1pt} V_{lm}\sigma _l^z\sigma _m^z$, where *σ* denote Pauli matrices, Γ_*l*_ (>0) are site-dependent transverse fields, and *V*_*lm*_ are arbitrary coupling constants.

**Fig. 2:**
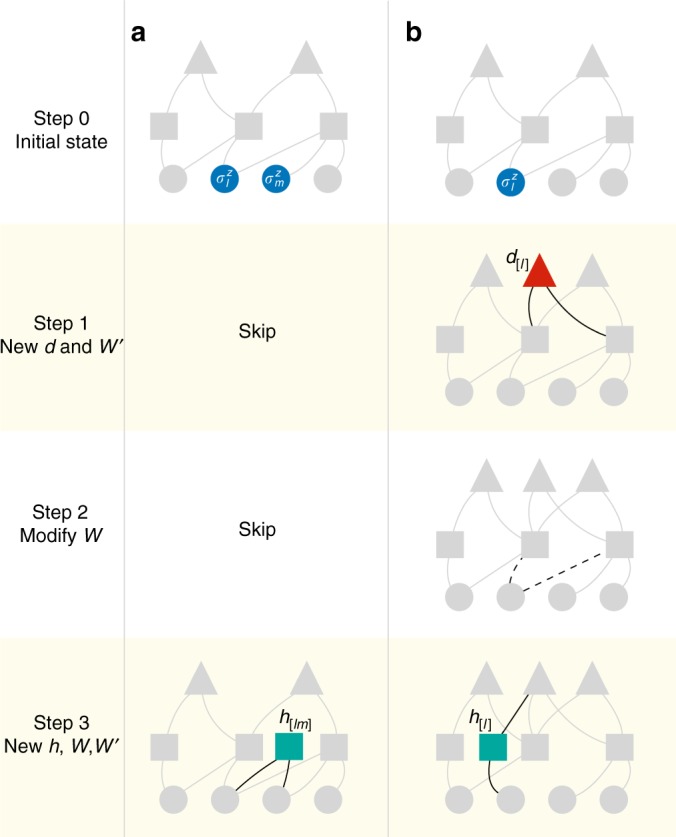
Construction of exact DBM representations of the transverse-field Ising model. In this example, a step of imaginary-time evolution is shown, for the case of the one-dimensional transverse-field Ising model. Dots represent physical degrees of freedom $\left( {\sigma _i^z} \right)$, squares represent hidden units (*h*_*j*_), triangles represent deep units (*d*_*k*_). In each panel, upper networks are the initial state with arbitrary network form, and the bottom networks are the final states, after application of the propagator. Intermediate steps illustrate how the network is modified, where the relevant modified couplings at each step are highlighted in black. The highlighted solid and dashed curves indicate new and vanishing couplings, respectively. **a** Shows the diagonal (interaction) propagator being applied to the highlighted blue spins. This introduces a hidden unit (green) connected only to the two physical spins. In (**b**) the off-diagonal (transverse-field) propagator is applied, acting on the blue physical spin. Here, we then add one deep unit (red triangle), and a hidden unit (green) mediating visible–deep interactions

In order to implement the mapping to a DBM, we first consider the action of the diagonal propagator ${\mathrm {e}}^{ - \delta _\tau V_{lm}\sigma _l^z\sigma _m^z}$, acting on a bond *V*_*lm*_. In this case, the goal of finding an exact DBM representation can be rephrased as finding solutions to

4 $$\left\langle {\sigma ^z} \right|{\mathrm {e}}^{ - \delta _\tau V_{lm}\sigma _l^z\sigma _m^z}\left| {\Psi _{\cal W}} \right\rangle = C{\mathrm{\Psi }}_{\bar {\cal W}}\left( {\sigma ^z} \right),$$

i.e. finding a set of new parameters $\bar {\cal W}$ that exactly reproduces the imaginary time evolution on the left- hand side. Here *C* is an arbitrary finite normalization constant. The diagonal propagator introduces an interaction between two visible, physical spins, which is not directly available in the DBM architecture. This interaction can be mediated by a new hidden unit in the first layer, *h*_[*lm*]_ which is only connected to the visible spins on that bond, i.e. $\bar W_{l[lm]}$ and $\bar W_{m[lm]}$ are finite, but $\bar W_{i[lm]}$ = 0, ∀*i* ≠ *l*, *m* and $\bar W_{j[lm]}^\prime$ = 0, ∀*j* (see Fig. 2a).

More concretely, the new wave function has then the form:

5 $$\begin{array}{c}{\mathrm{\Psi }}_{\bar {\cal W}}\left( {\sigma ^z} \right) = \mathop {\sum}\limits_{h_{[lm]}} {\kern 1pt} {\mathrm {e}}^{\sigma _l^zW_{l[lm]}h_{[lm]} + \sigma _m^zW_{m[lm]}h_{[lm]}}{\mathrm{\Psi }}_{\cal W}\left( {\sigma ^z} \right)\\ = 2{\kern 1pt} {\mathrm{cosh}}\left( {\sigma _l^zW_{l[lm]} + \sigma _m^zW_{m[lm]}} \right){\mathrm{\Psi }}_{\cal W}\left( {\sigma ^z} \right).\end{array}$$

Equation (4) is then satisfied if

6 $${\mathrm {e}}^{ - \delta _\tau V_{lm}\sigma _l^z\sigma _m^z} = 2C{\kern 1pt} {\mathrm{cosh}}\left( {\sigma _l^zW_{l[lm]} + \sigma _m^zW_{m[lm]}} \right)$$

for all the possible values of $\sigma _l^z$ and $\sigma _m^z$. By means of a useful identity [Eq. (21) in Methods], the new parameters *W*_*l*[*lm*]_ and *W*_*m*[*lm*]_ are given by

7 $$W_{l[lm]} = \frac{1}{2}{\mathrm{arcosh}}\left( {{\mathrm {e}}^{2\left| {V_{lm}} \right|\delta _\tau }} \right)$$

8 $$W_{m[lm]} = - {\mathrm{sgn}}\left( {V_{lm}} \right) \times W_{l[lm]}.$$

In this way the classical two-body interaction can, in general, be represented exactly by the shallow RBM.

Next, to exactly represent the off-diagonal propagator ${\mathrm {e}}^{\delta _\tau {\mathrm{\Gamma }}_l\sigma _l^x}\left| {{\mathrm{\Psi }}_{\cal W}} \right\rangle$, we must solve:

9 $$\begin{array}{r}{\mathrm{cosh}}\left( {{\mathrm{\Gamma }}_l\delta _\tau } \right){\mathrm{\Psi }}_{\cal W}\left( {\sigma ^z} \right) + {\mathrm{sinh}}\left( {{\mathrm{\Gamma }}_l\delta _\tau } \right){\mathrm{\Psi }}_{\cal W}\left( {\sigma _l^z \to - \sigma _l^z} \right)\\ = C{\mathrm{\Psi }}_{\bar {\cal W}}\left( {\sigma ^z} \right)\end{array}$$

for the new weights $\bar {\cal W}$, and for an appropriate finite normalization constant *C*. In this case, one possible solution is obtained by adding one deep *d*_[*l*]_ and one hidden *h*_[*l*]_ neurons. For *d*_[*l*]_, we create new couplings $W_{j[l]}^\prime$ to the existing hidden neurons *h*_*j*_ which are connected to $\sigma _l^z$. We simultaneously allow for changes in the existing parameters. By the procedure given in Methods, after applying the off-diagonal propagator for the site *l*, a solution of Eq. (9) is found by the matching condition of the hidden unit interactions on the left and the right hand sides of Eq. (9). Overall, the solution results in a three-step process (Fig. 2b): First, the hidden units attached to $\sigma _l^z$ are connected to the newly introduced deep unit *d*_[*l*]_ as

10 $$W_{j[l]}^\prime = - W_{lj}$$

(see Eq. (35)). Second, all the hidden units previously connected to the spin $\sigma _l^z$ lose their connection, i.e., $\bar W_{lj} = 0,\forall j$. Third, the spin $\sigma _l^z$ and the deep unit *d*_[*l*]_ are connected to the new hidden unit_,_*h*_[*l*]_, through the interaction*W*_*l*[*l*]_ and $W_{[l][l]}^\prime$, respectively, as

11 $$W_{l[l]} = \frac{1}{2}{\mathrm{arcosh}}\left( {\frac{1}{{{\mathrm{tanh}}\left( {{\mathrm{\Gamma }}_l\delta _\tau } \right)}}} \right),$$

12 $$W_{[l][l]}^\prime = - W_{l[l]}.$$

Using the given expressions for the parameters $\bar {\cal W}$ we can then exactly implement a single step of imaginary-time evolution. The full imaginary-time evolution is achieved by applying the above procedure for ${\cal H}_1$ and ${\cal H}_2$ alternately and repeatedly. Example applications of these rules, for both the diagonal and the off-diagonal propagators are shown in Fig. 2.

### Approximate RBM from DBM for transverse ising model

From the previous discussion, we have seen that the action of the off-diagonal propagator is responsible for the introduction of deep units in the network, thus breaking the shallow RBM structure. An interesting question is whether, in some limit, it is possible to stay within the RBM structure even for the off-diagonal propagator. The action of the off-diagonal propagator onto an RBM state can be then systematically expanded in powers of the weights:

13 $$	{\left\langle {\sigma ^z} \right|{\mathrm {e}}^{\delta _\tau {\mathrm{\Gamma }}_l\sigma _l^x}\left| {{\mathrm{\Psi }}_{\cal W}^{{\mathrm{RBM}}}} \right\rangle} \\ 	 {\propto \mathop {\sum}\limits_{\{ h\} } {\mathrm {e}}^{\mathop {\sum}\limits_{ij} {\kern 1pt} W_{ij}\sigma _i^zh_j}\left\{ {1 + {\mathrm{tanh}}\left( {{\mathrm{\Gamma }}_l\delta _\tau } \right)\left( {1 - 2\sigma _l^z\mathop {\sum}\limits_j h_jW_{lj}} \right)} \right\} + {\cal O}\left( {W_{lj}^2} \right).}$$

In the case of small weights, we can then exactly reproduce the off-diagonal propagator upon imposing a small change in the parameters *W*_*lj*_ → *W*_*lj*_ + Δ*W*_*lj*_ and keeping an RBM structure. If we expand the new RBM with modified weights, we get

14 $$\left\langle {\sigma ^z} \right|{\mathrm {e}}^{\delta _\tau {\mathrm{\Gamma }}_l\sigma _l^x}\left| {{\mathrm{\Psi }}_{\bar {\cal W}}^{{\mathrm{RBM}}}} \right\rangle \propto \mathop {\sum}\limits_{\{ h\} } {\mathrm {e}}^{\mathop {\sum}\limits_{ij} {\kern 1pt} W_{ij}\sigma _i^zh_j}\left\{ {1 + \sigma _l^z\mathop {\sum}\limits_j {\kern 1pt} {\mathrm{\Delta }}W_{lj}h_j} \right\} \\ \hskip 6pt \hskip 10pt + {\cal O}\left( {{\mathrm{\Delta }}W_{lj}^2} \right).$$

Comparing Eqs. (13) and (14), it follows that (apart from an irrelevant global normalization) the state after the off-diagonal propagator is still an RBM, with weights equal to:

15 $$W_{lj} \to W_{lj} - 2{\kern 1pt} {\mathrm{tanh}}\left( {{\mathrm{\Gamma }}_l\delta _\tau } \right)W_{lj},$$

and an error proportional to the square of the weights at that time step. In general, we expect that this kind of approximate updates is accurate in perturbative regimes (for example in the limit of small Γ_*l*_) or in the limit of small imaginary time evolution. A similar approximation scheme has been derived in ref. ^39^. Numerical results for this approximation are discussed in a dedicated section before the Discussion.

### Heisenberg model

We now consider the anti-ferromagnetic Heisenberg model (AFHM), on bipartite lattices. In one dimension, we decompose the Hamiltonian into odd and even bonds: ${\cal H}_1 = \mathop {\sum}\nolimits_{\langle l,m\rangle }^{{\mathrm{odd}}} {\kern 1pt} {\cal H}_{lm}^{{\mathrm{bond}}}$ and ${\cal H}_2 = \mathop {\sum}\nolimits_{\langle l,m\rangle }^{{\mathrm{even}}} {\cal H}_{lm}^{{\mathrm{bond}}}$, with ${\cal H}_{lm}^{{\mathrm{bond}}}$ = $J\left( {\sigma _l^x\sigma _m^x + \sigma _l^y\sigma _m^y + \sigma _l^z\sigma _m^z} \right)$, where *σ* denote Pauli matrices. Because the bond Hamiltonian ${\cal H}_{lm}^{{\mathrm{bond}}}$ is a building block also in higher dimensional models, construction of an exact DBM representation of the ground states can be achieved by finding solutions for the bond-propagator $\left\langle {\sigma ^z} \right|{\mathrm {e}}^{ - \delta _\tau {\cal H}_{lm}^{{\mathrm{bond}}}}\left| {{\mathrm{\Psi }}_{\cal W}} \right\rangle$ = $C\left\langle {\sigma ^z{\mathrm{|}}\Psi _{\bar {\cal W}}} \right\rangle$, where the parameters $\bar {\cal W}$ are such that the previous equation is satisfied for all the possible $\left\langle {\sigma ^z} \right|$, and for an arbitrary finite normalization constant *C*. More explicitly, we need to satisfy

16 $$\begin{array}{l}\delta _{\sigma _l^z,\sigma _m^z}{\mathrm {e}}^{ - J\delta _\tau }{\mathrm{\Psi }}_{\cal W}\left( {\sigma ^z} \right) + \left( {1 - \delta _{\sigma _l^z,\sigma _m^z}} \right){\mathrm {e}}^{J\delta _\tau }{\mathrm{cosh}}\left( {2J\delta _\tau } \right) \\ \times \left( {{\mathrm{\Psi }}_{\cal W}\left( {\sigma ^z} \right) - {\mathrm{tanh}}\left( {2J\delta _\tau } \right){\mathrm{\Psi }}_{\cal W}\left( {\sigma _l^z \leftrightarrow \sigma _m^z} \right)} \right) = C{\mathrm{\Psi }}_{\bar {\cal W}}\left( {\sigma ^z} \right).\end{array}$$

The basic strategy of finding a solution for Eq. (16) is similar to that for Eq. (9) in the transverse Ising model. Several possibilities arise when looking for solutions of the bond-propagator equation, Eq. (16). The existence of non-equivalent solutions prominently shows the non-uniqueness of DBM structure to represent the very same state and, at the same time, provides us flexibility in designing DBM architectures. Here, we show three concrete constructions. See Methods and Supplementary Note 2 for a detailed derivation of the DBM construction for the Heisenberg model, including anisotropic and bond-disordered coupling cases.

### 1 deep + 3 hidden variables construction for Heisenberg model

**Fig. 3:**
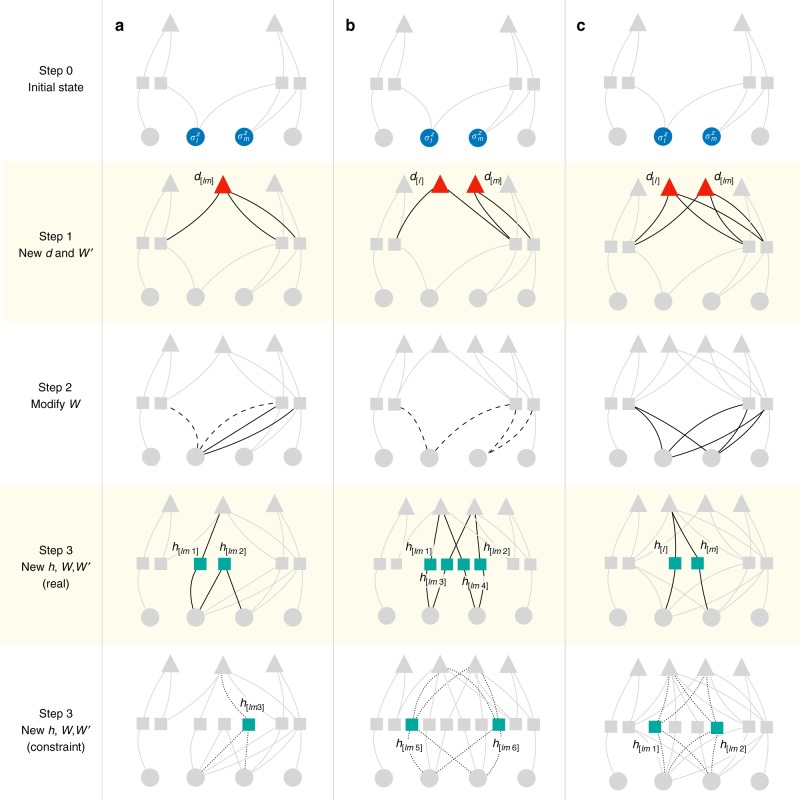
Construction of exact DBM representations of Heisenberg models. In this example, a time step of imaginary-time evolution is shown, for the case of the one-dimensional antiferromagnetic Heisenberg model. Dots represent physical degrees of freedom $\left( {\sigma _i^z} \right)$, squares represent hidden units (*h*_*j*_), triangles represent deep units (*d*_*k*_). The three panels (**a**–**c**) represent different possible explicit constructions. In each panel, upper networks are the initial state with arbitrary network form, and the bottom networks are the final states, after application of the propagator. Intermediate steps illustrate how the network is modified, where the relevant modified weights at each step are highlighted in black. In those diagrams, dashed lines indicate that the corresponding weights are set to zero, and dotted lines indicate complex-valued weights. The three panels correspond to the (**a**) “1 deep, 3 hidden” (1d–3h), (**b**) “2 deep, 6 hidden” (2d–6h), and (**c**) “2 deep, 4 hidden” (2d–4h) constructions (see text for a more detailed explanation of the individual steps characteristic of each construction)

The first construction is dubbed “1 deep, 3 hidden” (1d–3h). It amounts to adding an extra deep neuron, *d*_[*lm*]_, and three more hidden neurons to satisfy Eq. (16). A crucial difference with respect to the TFIM is that the introduced deep spin *d*_[*lm*]_ has a constraint depending on the state of the spins on the bond: $\sigma _l^z$ and $\sigma _m^z$. Specifically, when $\sigma _l^z = \sigma _m^z$ the deep spin is constrained to be $d_{[lm]} = \sigma _l^z = \sigma _m^z$, whereas when $\sigma _l^z \ne \sigma _m^z$, its value is unconstrained. From a pictorial point of view, the action of the bond propagator is a four-step process (see Fig. 3a). Starting from a given initial network (uppermost structures in Fig. 3), *d*_[*lm*]_ is added and connected, through $W_{j[lm]}^\prime$ given in Eq. (43), to the existing hidden units *h*_*j*_ connected to $\sigma _l^z$ and $\sigma _m^z$. Second, spin $\sigma _l^z$ is disconnected to all hidden units and reconnected to those hidden units the spin $\sigma _m^z$ is attached to [see Eq. (42)]. Third, two new hidden units are introduced. One of the hidden units, *h*_[*lm*1]_, mediates the interaction between $\sigma _l^z$ and *d*_[*lm*]_ [Eq. (47)], and the other hidden unit *h*_[*lm*2]_ mediates a direct spin–spin interaction between $\sigma _l^z$ and $\sigma _m^z$ [Eq. (49)]. Fourth, a further hidden unit connected to $\sigma _l^z$, $\sigma _m^z$, and *d*_[*lm*]_ is inserted, in such a way that the constraint previously described is satisfied. For all but the last step, the DBM weights are real-valued. In the last step instead the constraint is enforced by introducing imaginary-valued interactions (dotted lines in Fig. 3), referred to the “*iπ*/6‘ trick, resulting in a sign-problem-free global term ${\mathrm{cos}}({\pi {\mathrm{/}}6({\sigma _l^z + \sigma _m^z - d_{[lm]}})})$ after the summation over ±1 for the lastly added hidden unit *h*_[*lm*3]_: $\mathop {\sum}\nolimits_{h_{[lm3]} = \pm 1} {\kern 1pt} {\mathrm{exp}}[{i\pi {\mathrm{/}}6({\sigma _l^z + \sigma _m^z - d_{[lm]}})h_{[lm3]}}]$. The constraint mentioned above is assured by this cosine term.

### 2 deep + 6 hidden variables construction for Heisenberg model

The second construction is dubbed “2 deep, 6 hidden” (2d–6h), and is more similar to the lattice path-integral formulation. In this representation, we introduce two auxiliary deep spins per bond, *d*_[*l*]_ and *d*_[*m*]_ with constraint $d_{[l]} + d_{[m]} = \sigma _l^z + \sigma _m^z$, and six hidden neurons. The action of the bond propagator is schematically illustrated in Fig. 3b: first, two deep units *d*_[*l*]_ and *d*_[*m*]_ are introduced, connecting, respectively, to the hidden units spins $\sigma _l^z$ and $\sigma _m^z$ are attached to [see Eqs. (51) and (52)]. Second, all the connections between spins $\sigma _l^z$, $\sigma _m^z$, and hidden units *h*_*j*_ are cut off [Eqs. (53) and (54)]. Third, four hidden units *h*_[*lm*1]_, …, *h*_[*lm*4]_ are introduced, to mediate interactions between the two deep units and the physical spins *l*, *m* [Eqs. (61) and (62)]. Finally, two hidden units *h*_[*lm*5]_ and *h*_[*lm*6]_ are introduced, connecting both to *d*_[*l*]_, *d*_[*m*]_ and $\sigma _l^z,\sigma _m^z$ with imaginary-valued weights. The last step realizes the constraint $d_{[l]} + d_{[m]} = \sigma _l^z + \sigma _m^z$, through the “*iπ*/4, *iπ*/8‘ trick discussed in Methods and the discussion of the 2d–6h representation in Supplementary Note 2.

In this representation, if the hidden neurons are traced out, the imaginary-time evolution becomes equivalent to that of the path-integral Monte Carlo method. More specifically, the number of deep neurons introduced at each time slice is exactly the same as the number of visible spins, and the deep neurons at each time slice can be regarded as additional classical spin degrees of freedom in the path-integral. Moreover, the constraint $d_{[l]} + d_{[m]} = \sigma _l^z + \sigma _m^z$ ensures that the total magnetization is conserved at each time slice. Finally, the *W* and *W*′ interactions reproduce the matrix element of ${\mathrm{exp}}\left( { - \delta _\tau {\cal H}_{lm}^{{\mathrm{bond}}}} \right)$ between neighboring time slices. See Supplementary Note 2 for more detail on this point.

### 2 deep + 4 hidden variables construction for Heisenberg model

A further possible solution to Eq. (16) is dubbed “2 deep, 4 hidden” (2d–4h) construction. In this case, we introduce two auxiliary deep variables *d*_[*l*]_ and *d*_[*lm*]_. We also introduce four hidden units *h*_[*l*]_, *h*_[*m*]_, *h*_[*lm*1]_, and *h*_[*lm*2]_. Before the imaginary time evolution, $e^{ - \delta _\tau {\cal H}_{lm}^{{\mathrm{bond}}}}$, the physical variables $\sigma _n^z$ (*n* = *l* or *m*) are already coupled to each hidden variable *h*_*j*_ with a coupling *W*_*nj*_. After the time evolution ${\mathrm {e}}^{ - \delta _\tau {\cal H}_{lm}^{{\mathrm{bond}}}}$, as shown schematically in Fig. 3c, the coupling parameters are updated in the following way based on the old *W*_*nj*_: First, the first deep unit *d*_[*l*]_ becomes coupled to the already existing hidden variables *h*_*j*_ through the coupling $W_{j[l]}^\prime$ given in Eq. (67). The second deep unit *d*_[*lm*]_ becomes similarly coupled to *h*_*j*_ through a term *Z*_*lmj*_ given in Eq. (67). Second, *W*_*nj*_ is updated to $\bar W_{nj} = W_{nj} + {\mathrm{\Delta }}W_{nj}$ [see Eq. (66)]. Third, newly introduced *h*_[*n*]_ (*n* = *l* or *m*) gets coupled to *d*_[*l*]_ through $W_{[n][l]}^\prime$, and also to $\sigma _n^z$ through *W*_*n*[*n*]_ [Eqs. (71) and (73)].

Finally, as clarified in Methods, we also need to satisfy the constraint $d_{[l]}d_{[lm]} = \sigma _l^z\sigma _m^z$. Such a constraint is represented in DBM form as

17 $$\mathop {\sum}\limits_{h_{[lm1]},h_{[lm2]}} {\kern 1pt} {\mathrm{exp}}\left[ {\frac{{i\pi }}{4}\left( {h_{[lm1]} + h_{[lm2]}} \right)\left( {\sigma _l^z + \sigma _m^z + d_{[l]} + d_{[lm]}} \right)} \right],$$

which ensures $d_{[l]}d_{[lm]} = \sigma _l^z\sigma _m^z$ after explicit summation of *h*_[*lm*1]_ and *h*_[*lm*2]_. Finally, we remark that the three constructions presented here have different intrinsic network topologies. In particular, 2d–6h gives rise to a local topology (because of the equivalence with the path-integral contruction), 1d–3h has a local structure in the first layer and non-local in the second one, and 2d–4h is purely non-local in both layers.

### Sampling strategies

With network structures explicitly determined, we now focus on the problem of extracting meaningful physical quantities from them. To this end, it is convenient to decompose the DBM weight into two parts, such that

18 $${\mathrm{\Psi }}_{\cal W}\left( {\sigma ^z} \right) = \mathop {\sum}\limits_{\{ h,d\} } {\kern 1pt} P_1\left( {\sigma ^z,h} \right)P_2\left( {h,d} \right),$$

where $P_1\left( {\sigma ^z,h} \right)$ = ${\mathrm {e}}^{\sigma ^z \cdot a + \sigma ^z \cdot W \cdot h + h \cdot b}$, and $P_2(h,d)$ = ${\mathrm {e}}^{h \cdot W\prime \cdot d + d \cdot b\prime }$. The expectation value of an arbitrary (few-body) operator ${\cal O}$ can then be computed through the expression

19 $$\left\langle {\cal O} \right\rangle = \frac{{\mathop {\sum}\limits_{\left\{ {\sigma ^z,h,h\prime d,d\prime } \right\}} {\kern 1pt} {\mathrm{{\Pi}}}\left( {\sigma ^z,h,h\prime ,d,d\prime } \right)O_{{\mathrm{loc}}}\left( {\sigma ^z,h,h\prime } \right)}}{{\mathop {\sum}\limits_{\left\{ {\sigma ^z,h,h\prime d,d\prime } \right\}} {\mathrm{{\Pi}}}\left( {\sigma ^z,h,h\prime ,d,d\prime } \right)}},$$

where we have introduced the pseudo-probability density Π(*σ*^*z*^, *h*, *h*′, *d*, *d*′) ≡ $P_1\left( {\sigma ^z,h} \right)P_2\left( {h,d} \right)P_1^ \ast \left( {\sigma ^z,h\prime } \right)P_2^ \ast \left( {h\prime ,d\prime } \right)$, and the “local” estimator

20 $$O_{{\mathrm{loc}}}\left( {\sigma ^z,h,h\prime } \right) = \frac{1}{2}\mathop {\sum}\limits_{\sigma ^{\prime z}} \left\langle {\sigma ^z} \right|{\cal O}\left| {\sigma ^{\prime z}} \right\rangle \left( {\frac{{P_1\left( {\sigma ^{\prime z},h} \right)}}{{P_1\left( {\sigma ^z,h} \right)}} + \frac{{P_1\left( {\sigma ^{\prime z},h^\prime } \right)^ \ast }}{{P_1\left( {\sigma ^z,h^\prime } \right)^ \ast }}} \right).$$

For the sampling over the Π distribution, a block Gibbs sampling analogous to what performed in standard DBM architectures can be performed^37,40^. Alternatively, it is possible to devise a set of Metropolis local updates sampling the exactly known marginals ${\tilde{\mathrm {\Pi}}}\left( {\sigma ^z,h,h\prime } \right)$ = $\mathop {\sum}\nolimits_{\{ d,d\prime \} } {\kern 1pt} {\mathrm{{\Pi}}}\left( {\sigma ^z,h,h\prime ,d,d\prime } \right)$ or ${\tilde{\mathrm {\Pi}}}\prime \left( {\sigma ^z,d,d\prime } \right)$ = $\mathop {\sum}\nolimits_{\{ h,h\prime \} } {\mathrm{{\Pi}}}\left( {\sigma ^z,h,h\prime ,d,d\prime } \right)$.

In general, we have found that efficiently sampling the DBMs arising from the Heisenberg model constructions is typically more challenging than for the TFIM. This circumstance is a consequence of the imaginary couplings which set constraints on the value of hidden/deep units. These constraints typically make local Metropolis updates inefficient. With the notable exception of the 2d–6h representation, for which loop updates can be readily implemented, we leave the problem of designing efficient Monte Carlo sampling for the other Heisenberg constructions open. The sampling strategies adopted in our numerica are discussed more in detail in Supplementary Note 3.

### Numerical results

**Fig. 4:**
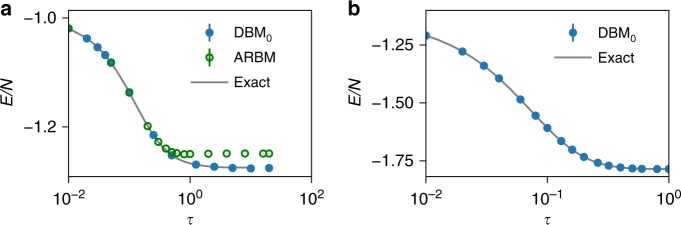
Imaginary-time evolution with a DBM for 1D spin models. **a** Expectation value of energy of the transverse-field Ising Hamiltonian in the exact imaginary-time evolution (continuous line) is compared to the stochastic result obtained with a DBM (filled circles) (*δ*_*τ*_ = 0.01). Empty circles correspond to the approximate RBM evolution scheme, Eq. (15). We consider the critical point (Γ_*l*_ = *V*_*lm*_), periodic boundary conditions, and *N* = 20 sites. **b** Expectation value of the isotropic antiferromagnetic Heisenberg Hamiltonian (AFHM) in the exact imaginary-time evolution (continuous line) is compared to the stochastic result obtained with a DBM (*δ*_*τ*_ = 0.01) following the 2d–6h construction. We consider periodic boundary conditions, *N* = 16 sites. The subscript *α* in DBM_*α*_ in panels (**a**, **b**) specifies a different initial state $\left| {{\mathrm{\Psi }}_0} \right\rangle$: *α* = 1 means that the initial state is an RBM state with hidden-unit density *M*/*N* = 1, whereas when *α* = 0 the initial state is the empty-network state (*M* = 0). All energies are in units of the transverse field (Γ_*l*_ = 1) for the TFIM, and of the exchange (*J* = 1) for the AFHM

We have implemented numerical algorithms to sample and obtain physical properties from the DBM previously derived. In Fig. 4a we show results for the one-dimensional TFIM. Specifically, we show the expectation value of the energy following the imaginary-time evolution starting from a fully polarized (in the *x* direction) initial state. The initial state corresponds to an empty network, where all the DBM parameters are set to zero. The DBM results closely match the exact imaginary-time evolution, thus verifying the correctness of our construction.

In Fig. 4a we also show the corresponding imaginary-time evolution as obtained from the approximate RBM construction, Eq. (15). As expected, this approximation is very accurate for short times, and breaks at later times.

**Fig. 5:**
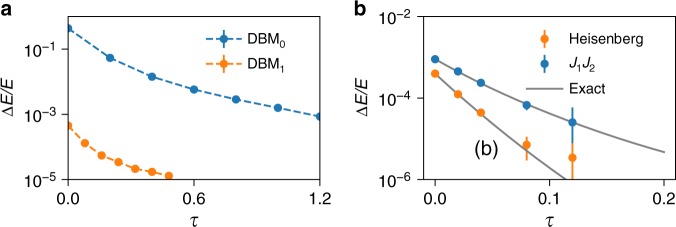
Approaching the exact ground-state energy. **a** Relative error on the ground-state energy for the 1D AFHM as a function of the imaginary time. Here we consider periodic boundary conditions, *N* = 80 sites, and *δ*_*τ*_ = 0.01, in units of the exchange *J* = 1. The subscript *α* in DBM_*α*_ specifies a different initial state $\left| {{\mathrm{\Psi }}_0} \right\rangle$: *α* = 1 means that the initial state is an RBM state with hidden-unit density *M*/*N* = 1, whereas when *α* = 0 the initial state is the empty-network state (*M* = 0). **b** Relative error on the ground-state energy for the two-dimensional *J*_1_ – *J*_2_ AFHM as a function of the imaginary time. As an energy unit, we consider *J*_1_ = 1, and take *J*_2_ = 0.0 and 0.4, periodic boundary conditions, *N* = 4×4 = 16 sites, and *δ*_*τ*_ = 0.001. Initial states are pre-optimized pair-product (geminal) state $\left| {\psi _{{\mathrm{PP}}}} \right\rangle$ supplemented by Gutzwiller factor $P_G^\infty$ = $\mathop {\prod}\nolimits_l \left( {1 - n_{l \uparrow }n_{l \downarrow }} \right)$ prohibiting double occupancy and quantum number projection onto the singlet state ${\cal L}^{S = 0}$, i.e., $\left| {{\mathrm{\Psi }}_0} \right\rangle$ = ${\cal L}^{S = 0}P_G^\infty \left| {\psi _{{\mathrm{PP}}}} \right\rangle$. The PP states are given by $\left| {\psi _{{\mathrm{PP}}}} \right\rangle$ = $\left( {\mathop {\sum}\nolimits_{l,m = 1}^N {\kern 1pt} f_{lm}^{ \uparrow \downarrow }c_{l \uparrow }^\dagger c_{m \downarrow }^\dagger } \right)^{N/2}\left| 0 \right\rangle$, where $f_{lm}^{ \uparrow \downarrow }$ are variational parameters and $c_{l\sigma }^\dagger$ are the operators creating the electron with spin *σ* at *l*th site

Numerical results for the one-dimensional Heisenberg model are shown in Figs. 4b and 5a. Specifically, 4b shows the numerical check for the DBM (construction 2d–6h) time evolution for one-dimensional Heisenberg model for *N* = 16. As expected, the DBM results also in this case follow the exact time evolution. Figure 5a shows the dependence of the energy from the initial state, for *N* = 80 case. Specifically, by taking a pre-optimized variational RBM as an initial state, we can significantly decrease the time *τ* needed to reach the ground state.

Results for two-dimensional models are shown in Fig. 5b, both for the two-dimensional Heisenberg model, and for the frustrated *J*_1_ − *J*_2_ model, on 4 × 4 lattice with periodic boundary conditions.

In the case of the TFIM, sampling from the DBM is realized through the Gibbs scheme previously sketched, in conjunction with a parallel tempering scheme, to improve ergodicity in the sampling.

For the AFHM and for the *J*_1_ − *J*_2_ model with 2d–6h representation, we adopt loop updates^41^ used in the path-integral QMC method, because the imaginary-time evolution in the 2d–6h representation has a direct correspondence to the path-integral formulation, allowing for an efficient handling of the constraint $d_{[l]} + d_{[m]}$ = $\sigma _l^z + \sigma _m^z$.

All the simulations carried here are sign-problem free, with the notable exception of the simulations carried on the two-dimensional *J*_1_ − *J*_2_ model. In this case, we start the imaginary-time evolution from a pre-optimized variational wave function, thus setting the fully evolved state as product of a DBM and the initial state. Because of the quality of the initial guess, a moderate sign problem can be numerically afforded for short time evolutions, and in this case it is enough to converge to the exact ground state (see Fig. 5b).

## Discussion

We have shown how exact ground states of interacting spin Hamiltonians can be explicitly constructed using artificial neural networks comprising only two layers of hidden variables. In contrast to approaches based on one-layer RBMs, the constructions we have derived here do not require further variational optimization of the network parameters, and the exact representation of many-body ground states can be achieved with only polynomially many neurons. In the case of the Heisenberg model, all of the explicit algorithms presented here give rise to sign-problem-free representations, if the lattice is bipartite.

The DBM representation has an intrinsic conceptual value, as an alternative quantum-to-classical mapping to the path-integral representation. In the path-integral formalism, the addition of an extra dimension (the imaginary time direction) is needed to exactly represent the quantum many-body state. In our case, the DBM deep hidden layer plays a similar role as the additional dimension in the path integral. As argued in Methods [see Eq. (28)], a single-layer RBM is indeed sufficient to exactly, and efficiently describe the state of arbitrary classical spin systems. On the other hand, a second, deep layer is necessary for the efficient, and exact construction of compact networks describing quantum mechanical states.

DBM-based schemes can be further used to systematically improve upon existing RBM variational results. More generally, the initial state for the present DBM scheme can be generic variational states or even combinations of RBMs and more conventional wave functions^24,33^. We have shown that, by starting the DBM construction from a pre-optimized variational state, a fast convergence to the exact ground state is observed. As shown in Fig. 5b, this kind of scheme opens the possibility of characterizing the ground state even in the case of non-bipartite lattices with frustration effects, exploiting the transient regime in which the sign problem can be still efficiently handled numerically, as for example discussed in ref. ^42^.

## Methods

### Useful identities

It is useful to introduce several identities, which can be used when more complicated interactions between the visible spins *σ*^*z*^, hidden variables *h* and deep variables *d* beyond the standard form Eq. (1) are needed. The first identity reads

21 $${\mathrm {e}}^{s_1s_2V} = C\mathop {\sum}\limits_{s_3 = \pm 1} {\kern 1pt} {\mathrm {e}}^{s_1s_3\tilde V_1 + s_2s_3\tilde V_2} = 2C\,{\mathrm{cosh}}\left( {s_1\tilde V_1 + s_2\tilde V_2} \right).$$

with

22 $$C = \frac{1}{2}{\mathrm {e}}^{ - |V|}$$

23 $$\tilde V_1 = \frac{1}{2}{\mathrm{arcosh}}\left( {{\mathrm {e}}^{2|V|}} \right)$$

24 $$\tilde V_2 = {\mathrm{sgn}}(V) \times \tilde V_1$$

for Ising variables *s*_1_ and *s*_2_, and a real interaction *V*. This is a gadget for decomposing two-body interactions, and can be proven by examining all the cases of *s*_1_ and *s*_2_.

By taking *s*_1_ and *s*_2_ as visible (physical) variables *σ*^*z*^ and *s*_3_ as a hidden variable *h*, the direct classical two-body interaction between physical variables [the leftmost part in Eq. (21)] is cut and instead mediated by the hidden neuron *h*. Furthermore, a direct interaction between *σ*^*z*^ and *d* can also be decomposed: In the following derivations for the DBM wave constructions, for convenience, we sometimes introduce the direct interaction between *σ*^*z*^ and *d*, which is not allowed in the DBM structure. However, by taking *s*_1_ as a visible spin *σ*^*z*^, *s*_2_ as a deep variable *d*, and *s*_3_ as a hidden variable *h* in Eq. (21), one can eliminate the direct interaction between *σ*^*z*^ and *d* and decompose it into the interaction mediated only by *h* with trade-off of the summation over the hidden variable *h*. With this trick, one can recover the standard DBM form in Eq. (1).

Another identity (decomposition of four-body interaction) is

25 $$\begin{array}{c}{\mathrm {e}}^{s_1s_2s_3s_4V} = \frac{1}{4}\mathop {\sum}\limits_{s_5,s_6,s_7} {\kern 1pt} {\mathrm{exp}}\left[ {i\frac{\pi }{4}\left( {s_5 + s_6} \right)\left( {s_1 + s_2 + s_3 + s_7} \right)} \right]\\ \times {\mathrm{exp}}\left( {s_4s_7V} \right)\\ = \mathop {\sum}\limits_{s_7} {\kern 1pt} {\mathrm{cos}}^2\left[ {\frac{\pi }{4}\left( {s_1 + s_2 + s_3 + s_7} \right)} \right]{\mathrm{exp}}\left( {s_4s_7V} \right)\end{array}$$

for Ising variables *s*_*i*_ with *i* = 1,…, 4. Although we have introduced complex couplings in the first line, each term in the summation in the second line of Eq. (25) is positive definite if *V* is real. The second line remains nonzero only if *s*_1_*s*_2_ = *s*_3_*s*_7_, which proves the identity. This identity with *s*_1_ and *s*_2_ as physical variables, *s*_4_, *s*_5_, and *s*_6_ as hidden variables, and *s*_3_ and *s*_7_ as deep variables, reads

26 $$\begin{array}{c}{\mathrm {e}}^{\sigma _1\sigma _2d_1h_1V} = \frac{1}{4}\mathop {\sum}\limits_{h_2,h_3,d_2} {\kern 1pt} {\mathrm{exp}}\left[ {i\frac{\pi }{4}\left( {h_2 + h_3} \right)\left( {\sigma _1 + \sigma _2 + d_1 + d_2} \right)} \right]\\ \times {\mathrm{exp}}\left( {h_1d_2V} \right),\end{array}$$

Note that the right-hand side fits the DBM structure.

General three-body and two-body interactions can also be represented by the two-body form just by putting some of *s*_1_,…,*s*_4_ as constants in Eq. (25). These could be used instead of Eq. (21), although we employ Eq. (21) in the formalism below for the decoupling of the two-body interaction.

Finally, we discuss the gadgets for decomposing general *N*-body classical interactions using complex bias term *b*_*j*_ in addition to the couplings *W* and *W*′, whereas the gadgets Eqs. (21) and (26) are represented only by *W* and *W*′ interactions. The gadget reads

27 $${\mathrm {e}}^{\sigma _1\sigma _2 \ldots \sigma _NV} = C\,{\mathrm{cos}}^2\left( {b + \frac{\pi }{4}\mathop {\sum}\limits_{i = 1}^N {\kern 1pt} \sigma _i} \right)$$

28 $$= \frac{C}{4}\mathop {\sum}\limits_{h_1,h_2} {\kern 1pt} {\mathrm {e}}^{ib\left( {h_1 + h_2} \right)}{\mathrm {e}}^{i\frac{\pi }{4}\left( {h_1 + h_2} \right)\left( {\sigma _1 + \sigma _2 + \ldots + \sigma _N} \right)}$$

with

29 $$b = {\mathrm{arctan}}\left( {{\mathrm {e}}^{ - V}} \right) - \frac{\pi }{4}{\mathrm{mod}}\left( {N,4} \right),$$

30 $$C = \frac{1}{{{\mathrm{cos}}\left( {{\mathrm{arctan}}\left( {{\mathrm {e}}^{ - V}} \right)} \right) \times {\mathrm{sin}}\left( {{\mathrm{arctan}}\left( {{\mathrm {e}}^{ - V}} \right)} \right).}}$$

This fact suggests that any classical partition function defined for Ising spins can be written exactly in terms of an RBM. Although the RBM is shown to be powerful in representing also the quantum states, there is no analytical way to map quantum states to the RBM and one must rely on numerical optimizations to get the RBM parameters. In the present study, we show analytical mappings from quantum states to the DBM, which has additional hidden layer. In the statistical mechanics, it is known that quantum systems with *D* dimension can be mapped on (*D* + 1)-dimensional classical systems. Therefore, having additional hidden layer in neural network language is equivalent to acquiring additional dimension in statistical mechanics.

### Transverse-field ising model

The solution of Eq. (9) is found in the following way. The left-hand side of Eq. (9) can be rewritten by using the notation Eq. (18) as

31 $$\begin{array}{l}\mathop {\sum}\limits_{\{ h,d\} } {\kern 1pt} P_1\left( {\sigma ^z,h} \right)P_2(h,d)\left[ {1 + {\mathrm{tanh}}\left( {{\mathrm{\Gamma }}_l\delta _\tau } \right){\mathrm {e}}^{ - 2\sigma _l^z\mathop {\sum}\limits_j {\kern 1pt} h_jW_{lj}}} \right]\\ = C{\mathrm{\Psi }}_{\bar {\cal W}}\left( {\sigma ^z} \right).\end{array}$$

We look for a solution by adding one deep neuron *d*_[*l*]_ and creating new couplings $W_{j[l]}^\prime$ to the existing hidden neurons *h*_*j*_ which are connected to $\sigma _l^z$. We also allow for changes in the existing interaction parameters. In particular we set the new couplings to be $\bar W_{lj} = W_{lj} + {\mathrm{\Delta }}W_{lj}$, (with Δ*W*_*lj*_ to be determined). Moreover, we introduce one hidden neuron *h*_[*l*]_ coupled to $\sigma _l^z$ and *d*_[*l*]_ through the interactions *W*_*l*[*l*]_ and $W_{[l][l]}^\prime$, respectively. If we trace out *h*_[*l*]_, the hidden neuron *h*_[*l*]_ mediates the interaction between $\sigma _l^z$ and *d*_[*l*]_ (denoted as $W_{l[l]}^{\prime\prime}$).

With this choice, we have (in the representation where *h*_[*l*]_ is traced out):

32 $$\begin{array}{*{20}{l}} {{\mathrm{\Psi }}_{\bar {\cal W}}\left( {\sigma ^z} \right)} \hfill & = \hfill & {\mathop {\sum}\limits_{\{ h,d\} } \mathop {\sum}\limits_{d_{[l]}} {\kern 1pt} P_1\left( {\sigma ^z,h} \right)P_2(h,d)} \hfill \\ {} \hfill & {} \hfill & {{\mathrm {e}}^{\sigma _l^z\mathop {\sum}\limits_j {\kern 1pt} {\mathrm{\Delta }}W_{lj}h_j + d_{[l]}\mathop {\sum}\limits_j {\kern 1pt} h_jW_{j[l]}^\prime + \sigma _l^zd_{[l]}W_{l[l]}^{\prime\prime} }.} \hfill \end{array}$$

The equations to be verified are obtained considering the two possible values of $\sigma _l^z = \pm 1$:

33 $${{\mathrm {e}}^{\mathop {\sum}\limits_j \,h_j\left( {{\mathrm{\Delta }}W_{lj} + W_{j[l]}^\prime } \right) + W_{l[l]}^{\prime\prime} } + {\mathrm {e}}^{\mathop {\sum}\limits_j {\kern 1pt} h_j\left( {{\mathrm{\Delta }}W_{lj} - W_{j[l]}^\prime } \right) - W_{l[l]}^{\prime\prime} } = C \times \left( {1 + {\mathrm{tanh}}\left( {{\mathrm{\Gamma }}_l\delta _\tau } \right){\mathrm {e}}^{ - 2\mathop {\sum}\limits_j {\kern 1pt} h_jW_{lj}}} \right)}$$

34 $${{\mathrm {e}}^{\mathop {\sum}\limits_j {\kern 1pt} h_j\left( { - {\mathrm{\Delta }}W_{lj} + W_{j[l]}^\prime } \right) - W_{l[l]}^{\prime\prime} } + {\mathrm {e}}^{\mathop {\sum}\limits_j {\kern 1pt} h_j\left( { - {\mathrm{\Delta }}W_{lj} - W_{j[l]}^\prime } \right) + W_{l[l]}^{\prime\prime} } = C \times \left( {1 + {\mathrm{tanh}}\left( {{\mathrm{\Gamma }}_l\delta _\tau } \right){\mathrm {e}}^{2\mathop {\sum}\limits_j {\kern 1pt} h_jW_{lj}}} \right).}$$

This equation has a solution from the requirement that the hidden unit interactions on the left and right hand sides match, thus we require

35 $${\mathrm{\Delta }}W_{lj} + W_{j[l]}^\prime = - 2W_{lj}$$

36 $${\mathrm{\Delta }}W_{lj} - W_{j[l]}^\prime = 0,$$

and

37 $$W_{l[l]}^{\prime\prime} = \frac{{{\mathrm{log}} \, {\mathrm{tanh}}\left( {{{\Gamma }}_{l}\delta _{\tau} } \right)}}{2}.$$

Notice that when Γ_*l*_ > 0, $W_{l[l]}^{\prime\prime}$ is also real. By using Eq. (21) with the following replacement $s_1 \to \sigma _l^z$, *s*_2_ → *d*_[*l*]_, *s*_3_ → *h*_[*l*]_, $V \to W_{l[l]}^{\prime\prime}$, $\tilde V_1 \to W_{l[l]}$, and $\tilde V_2 \to W_{[l][l]}^\prime$, the last condition determines the real couplings *W*_*l*[*l*]_ and $W_{[l][l]}^\prime$ as Eqs. (11) and (12).

### Heisenberg model

Here, we show the derivation for the general form of bond Hamiltonian allowing anisotropy and bond-disorder: ${\cal H}_{lm}^{{\mathrm{bond}}}$ = $J_{lm}^{xy}\left( {\sigma _l^x\sigma _m^x + \sigma _l^y\sigma _m^y} \right)$ + $J_{lm}^z\sigma _l^z\sigma _m^z$. In the case of the bipartite lattice and the antiferromagnetic exchange $J_{lm}^z,J_{lm}^{xy} > 0$, we further apply a local gauge transformation by a *π* rotation around the *z*-axis in the spin space as *σ*^*x*^ → −*σ*^*x*^ and *σ*^*y*^ → −*σ*^*y*^ on one of the sublattices, which gives a – sign for $\sigma _l^x\sigma _m^x$ and $\sigma _l^y\sigma _m^y$ interactions. This transformation is equivalent to taking

38 $$J_{lm}^{xy} \to - J_{lm}^{xy}.$$

The gauge transformation enables to design a DBM neural network with real couplings {*W*, *W*′} except for those to put “constraint‘ on the values of deep neuron spins (see more detail about the constraint in the following sections). It ensures that the DBM algorithm has no negative sign problems.

In the case of the antiferromagnetic Heisenberg model after the gauge transformation on the bipartite lattice, we must solve, for each bond,

39 $$\begin{array}{l}\delta _{\sigma _l^z,\sigma _m^z}{\mathrm {e}}^{ - \delta _\tau J_{lm}^z}{\mathrm{\Psi }}_{\cal W}\left( {\sigma ^z} \right) + \left( {1 - \delta _{\sigma _l^z,\sigma _m^z}} \right){\mathrm {e}}^{\delta _\tau J_{lm}^z}\\ \left( {{\mathrm{\Psi }}_{\cal W}\left( {\sigma ^z} \right){\mathrm{cosh}}\left( {2J_{lm}^{xy}\delta _\tau } \right) + {\mathrm{\Psi }}_{\cal W}\left( {\sigma _l^z \leftrightarrow \sigma _m^z} \right){\mathrm{sinh}}\left( {2J_{lm}^{xy}\delta _\tau } \right)} \right)\\ = C\left\langle {\sigma ^z{\mathrm{|\Psi }}_{\bar {\cal W}}} \right\rangle .\end{array}$$

It is also useful to explicitly write the expression for the exchange term in the second line above:

40 $$\begin{array}{r}{\mathrm{\Psi }}_{\cal W}\left( {\sigma ^z} \right){\mathrm{cosh}}\left( {2J_{lm}^{xy}\delta _\tau } \right) + {\mathrm{\Psi }}_{\cal W}\left( {\sigma _l^z \leftrightarrow \sigma _m^z} \right){\mathrm{sinh}}\left( {2J_{lm}^{xy}\delta _\tau } \right)\\ = \mathop {\sum}\limits_{\{ h,d\} } {\kern 1pt} P_1\left( {\sigma ^z,h} \right)P_2(h,d)\left[ {{\mathrm{cosh}}\left( {2J_{lm}^{xy}\delta _\tau } \right) } \right.\\ \left. +{{\mathrm{sinh}}\left( {2J_{lm}^{xy}\delta _\tau } \right)e^{\left( {\sigma _m^z - \sigma _l^z} \right)\mathop {\sum}\limits_j {\kern 1pt} h_j\left( {W_{lj} - W_{mj}} \right)}} \right].\end{array}$$

In the following derivations, for the antiferromagnetic Hamiltonian $\left( {J_{lm}^z,J_{lm}^{xy} \,> \,0} \right)$ after the gauge transformation, we look for a solution with zero bias terms ($a_i,b_j,b_k^\prime = 0$, ∀*i*, *j*, *k*). We can also derive a sign-problem-free solution for the imaginary time evolution in the absence of the explicit gauge transformation by introducing a complex bias term *a*_*i*_. Indeed, in the “2 deep, 4 hidden” representation, we will explicitly show that taking a specific set of complex bias term *a*_*i*_ on physical spins is equivalent to the gauge transformation, making a solution free from the sign problem.

In a way similar to the TFIM, solutions of Eq. (39) can be found by specifying the structure of the DBM and the three examples are the following.

### 1d–3h construction for Heisenberg model

We assume the structure of the updated wave function (corresponding to Eq. (32) for the TFIM) to be

41 $$\begin{array}{l}{\mathrm{\Psi }}_{\bar {\cal W}}\left( {\sigma ^z} \right) = \mathop {\sum}\limits_{\{ h,d\} } \mathop {\sum}\limits_{{\begin{array}{*{20}{c}} {d_{[lm]} = \pm 1} \\ {d_{[lm]} = \sigma _l^z\;{\mathrm{if}}\;\sigma _l^z = \sigma _m^z} \end{array}}} P_1\left( {\sigma ^z,h} \right)P_2(h,d)\\ {\mathrm {e}}^{\sigma _l^z\mathop {\sum}\limits_j {\kern 1pt} {\mathrm{\Delta }}W_{lj}h_j + d_{[lm]}\mathop {\sum}\limits_j h_jW_{j[lm]}^\prime + d_{[lm]}\sigma _l^zW_{l[lm]}^{\prime\prime} + V_{[lm]}\sigma _l^z\sigma _m^z}.\end{array}$$

Similarly to the case of the TFIM, a solution of Eq. (39) is given by

42 $${\mathrm{\Delta }}W_{lj} = - W_{lj} + W_{mj}$$

43 $$W_{j[lm]}^\prime = W_{lj} - W_{mj}.$$

and

44 $$W_{l[lm]}^{\prime\prime} = - \left( {{\mathrm{log}} \, {\mathrm{tanh}}\left( {2J_{lm}^{xy}\delta _\tau } \right)} \right){\mathrm{/}}2$$

45 $$V_{[lm]} = - \left( {{\mathrm{log}} \, {\mathrm{cosh}}\left( {2J_{lm}^{xy}\delta _\tau } \right)} \right){\mathrm{/}}2 - J_{lm}^z\delta _\tau$$

Notice that the first condition is equivalent to cutting all connections from spin *l* to the hidden units and attaching the spin *l* to all the hidden units connected to spin *m*, with an interaction *W*_*mj*_.

Although the terms proportional to $W_{l[lm]}^{\prime\prime}$ and *V*_*lm*_ do not satisfy the standard DBM form, they can be transformed to the DBM form by introducing new hidden neurons *h*_[*lm*1]_ and *h*_[*lm*2]_ [see the gadget Eq. (21)]:

46 $${\mathrm {e}}^{\sigma _l^zd_{[lm]}W_{l[lm]}^{\prime\prime} } = C_{[lm1]}\mathop {\sum}\limits_{h_{[lm1]}} {\kern 1pt} {\mathrm {e}}^{\sigma _l^zh_{[lm1]}W_{l[lm1]} + h_{[lm1]}d_{[lm]}W_{[lm1][lm]}^\prime },$$

with

47 $$W_{l[lm1]} = W_{[lm1][lm]}^\prime = \frac{1}{2}{\mathrm{arcosh}}\left( {\frac{1}{{{\mathrm{tanh}}\left( {2J_{lm}^{xy}\delta _\tau } \right)}}} \right).$$

Similarly, the coupling *V*_[*lm*]_ is decomposed as

48 $${\mathrm {e}}^{\sigma _l^z\sigma _m^zV_{[lm]}} = C_{[lm2]}\mathop {\sum}\limits_{h_{[lm2]}} {\kern 1pt} {\mathrm {e}}^{\sigma _l^zh_{[lm2]}W_{l[lm2]} + \sigma _m^zh_{[lm2]}W_{m[lm2]}},$$

with

49 $$W_{l[lm2]} = - W_{m[lm2]} = \frac{1}{2}{\mathrm{arcosh}}\left( {{\mathrm{cosh}}\left( {2J_{lm}^{xy}\delta _\tau } \right)e^{2J_{lm}^z\delta _\tau }} \right).$$

Finally, as discussed in the main text, the constraint $d_{[lm]} = \sigma _l^z$ when $\sigma _l^z = \sigma _m^z$ can be satisfied by adding the third neuron *h*_[*lm*3]_, introducing pure complex *iπ*/6 couplings.

### 2d–6h construction for Heisenberg model

In this case, the form of the new wave function reads

50 $$\begin{array}{l}\Psi _{\bar {\cal W}}\left( {\sigma ^z} \right) = \mathop {\sum}\limits_{\{ h,d\} } \mathop {\sum}\limits_{\begin{array}{*{20}{c}} {d_{[l]},d_{[m]}} \\ {d_{[l]} + d_{[m]} = \sigma _l^z + \sigma _m^z} \end{array}} P_1\left( {\sigma ^z,h} \right)P_2(h,d)\\ {\mathrm {e}}^{\mathop {\sum}\limits_j \mathop {\sum}\limits_{n = l,m} {\kern 1pt} h_j\left( {{\mathrm{\Delta }}W_{nj}\sigma _n^z + W_{j[n]}^\prime d_{[n]}} \right) + \mathop {\sum}\limits_{n = l,m} \sigma _n^z\left( {W_{n[l]}^{\prime\prime} d_{[l]} + W_{n[m]}^{\prime\prime} d_{[m]}} \right)}.\end{array}$$

A solution of Eq. (39) is given by

51 $$W_{j[l]}^\prime = W_{lj},$$

52 $$W_{j[m]}^\prime = W_{mj},$$

53 $${\mathrm{\Delta }}W_{lj} = - W_{lj},$$

54 $${\mathrm{\Delta }}W_{mj} = - W_{mj},$$

and

55 $$W_{l[l]}^{\prime\prime} = W_{m[m]}^{\prime\prime} = - \frac{{J_{lm}^z\delta _\tau }}{2} - \frac{1}{4}{\mathrm{log}} \, {\mathrm{sinh}}\left( {2J_{lm}^{xy}\delta _\tau } \right),$$

56 $$W_{l[m]}^{\prime\prime} = W_{m[l]}^{\prime\prime} = - \frac{{J_{lm}^z\delta _\tau }}{2} - \frac{1}{4}{\mathrm{log}} \, {\mathrm{ cosh}}\left( {2J_{lm}^{xy}\delta _\tau } \right).$$

The direct interactions between $\left( {\sigma _l^z,d_{[l]}} \right)$, $\left( {\sigma _m^z,d_{[m]}} \right)$, $\left( {\sigma _l^z,d_{[m]}} \right)$, and $\left( {\sigma _m^z,d_{[l]}} \right)$, are mediated by *h*_[*lm*1]_, *h*_[*lm*2]_, *h*_[*lm*3]_, and *h*_[*lm*4]_, respectively, as follows:

57 $$\begin{array}{c}{\mathrm {e}}^{\sigma _l^zd_{[l]}W_{l[l]}^{\prime\prime} } = C_{[lm1]} \mathop {\sum}\limits_{h_{[lm1]}} {\kern 1pt} {\mathrm {e}}^{\sigma _l^zh_{[lm1]}W_{l[lm1]} + h_{[lm1]}d_{[l]}W_{[lm1][l]}^\prime },\end{array}$$

58 $$\begin{array}{c}{\mathrm {e}}^{\sigma _m^zd_{[m]}W_{m[m]}^{\prime\prime} } = C_{[lm2]} \mathop {\sum}\limits_{h_{[lm2]}} {\kern 1pt} {\mathrm {e}}^{\sigma _m^zh_{[lm2]}W_{m[lm2]} + h_{[lm2]}d_{[m]}W_{[lm2][m]}^\prime },\end{array}$$

59 $$\begin{array}{c}{\mathrm {e}}^{\sigma _l^zd_{[m]}W_{l[m]}^{\prime\prime} } = C_{[lm3]} \mathop {\sum}\limits_{h_{[lm3]}} {\kern 1pt} {\mathrm {e}}^{\sigma _l^zh_{[lm3]}W_{l[lm3]} + h_{[lm3]}d_{[m]}W_{[lm3][m]}^\prime },\end{array}$$

60 $$\begin{array}{c}{\mathrm {e}}^{\sigma _m^zd_{[l]}W_{m[l]}^{\prime\prime} } = C_{[lm4]} \mathop {\sum}\limits_{h_{[lm4]}} {\kern 1pt} {\mathrm {e}}^{\sigma _m^zh_{[lm4]}W_{m[lm4]} + h_{[lm4]}d_{[l]}W_{[lm4][l]}^\prime }.\end{array}$$

By applying the gadget Eq. (21), the new *W* and *W*′ interactions are given by, for small *δ*_*τ*_ (such that ${\textstyle{{{\mathrm {e}}^{ - J_{lm}^z\delta _\tau }} \over {\sqrt {{\mathrm{sinh}}\left( {2J_{lm}^{xy}\delta _\tau } \right)} }}} > 1$),

61 $$\begin{array}{c}W_{l[lm1]} = W_{[lm1][l]}^\prime = W_{m[lm2]} = W_{[lm2][m]}^\prime \\ = \frac{1}{2}{\mathrm{arcosh}}\left( {\frac{{{\mathrm {e}}^{ - J_{lm}^z\delta _\tau }}}{{\sqrt {{\mathrm{sinh}}\left( {2J_{lm}^{xy}\delta _\tau } \right)} }}} \right)\end{array}$$

and

62 $$\begin{array}{c}W_{l[lm3]} = - W_{[lm3][m]}^\prime = W_{m[lm4]} = - W_{[lm4][l]}^\prime \\ = \frac{1}{2}{\mathrm{arcosh}}\left( {\sqrt {{\mathrm{cosh}}\left( {2J_{lm}^{xy}\delta _\tau } \right)} \times {\mathrm {e}}^{J_{lm}^z\delta _\tau }} \right).\end{array}$$

Finally, the constraint $d_{[l]} + d_{[m]} = \sigma _l^z + \sigma _m^z$ can be put by introducing additionally two hidden neurons *h*_[*lm*5]_ and *h*_[*lm*6]_, and by introducing complex couplings

63 $$\begin{array}{r}\mathop {\sum}\limits_{h_{[lm5]},h_{[lm6]}} {\kern 1pt} {\mathrm {e}}^{i\frac{\pi }{4}\left( {\left( {\sigma _l^z + \sigma _m^z} \right)h_{[lm5]} - h_{[lm5]}\left( {d_{[l]} + d_{[m]}} \right)} \right)}\\ \times {\mathrm {e}}^{i\frac{\pi }{8}\left( {\left( {\sigma _l^z + \sigma _m^z} \right)h_{[lm6]} - h_{[lm6]}\left( {d_{[l]} + d_{[m]}} \right)} \right)}\end{array}$$

This term gives interactions among *d*_[*l*]_, *d*_[*m*]_, $\sigma _l^z$ and $\sigma _m^z$: $4\,{\mathrm{cos}}\left( {{\textstyle{\pi \over 4}}\left( {\sigma _l^z + \sigma _m^z - d_{[l]} - d_{[m]}} \right)} \right)$ ${\mathrm{cos}}\left( {{\textstyle{\pi \over 8}}\left( {\sigma _l^z + \sigma _m^z - d_{[l]} - d_{[m]}} \right)} \right)$, which realize the constraint.

### 2d–4h construction for Heisenberg model

For this construction, we assume the following structure for the wave-function after the propagator:

64 $$\begin{array}{c}{\mathrm{\Psi }}_{\bar {\cal W}}\left( {\sigma ^z} \right) = \mathop {\sum}\limits_{\{ h,d\} } \mathop {\sum}\limits_{d_{[l]}} {\kern 1pt} P_1\left( {\sigma ^z,h} \right)P_2(h,d){\mathrm {e}}^{\mathop {\sum}\limits_{j,n = l,m} {\kern 1pt} \sigma _n^zh_j{\mathrm{\Delta }}W_{nj}}\\ \times {\mathrm {e}}^{\mathop {\sum}\limits_j {\kern 1pt} h_jd_{[l]}W_{j[l]}^\prime + \mathop {\sum}\limits_{n = l,m} {\kern 1pt} \sigma _n^zd_{[l]}W_{n[l]}^{\prime\prime} + \mathop {\sum}\limits_j {\kern 1pt} \sigma _l^z\sigma _m^zh_jd_{[l]}Z_{lmj}}.\end{array}$$

In this case, we also look for a solution for the bond operator without the gauge transformation. This shows that the introduction of a complex bias term *a*_*i*_ can play the same role as the gauge transformation. Then, we need to solve

65 $$\begin{array}{c}\delta _{\sigma _l^z,\sigma _m^z}{\mathrm {e}}^{ - \delta _\tau J_{lm}^z}{\mathrm{\Psi }}_{\cal W}\left( {\sigma ^z} \right) + \left( {1 - \delta _{\sigma _l^z,\sigma _m^z}} \right){\mathrm {e}}^{\delta _\tau J_{lm}^z}\\ \left( {{\mathrm{\Psi }}_{\cal W}\left( {\sigma ^z} \right){\mathrm{cosh}}\left( {2J_{lm}^{xy}\delta _\tau } \right) - {\mathrm{\Psi }}_{\cal W}\left( {\sigma _l^z \leftrightarrow \sigma _m^z} \right){\mathrm{sinh}}\left( {2J_{lm}^{xy}\delta _\tau } \right)} \right)\\ = C\left\langle {\sigma ^z{\mathrm{|\Psi }}_{\bar {\cal W}}} \right\rangle .\end{array}$$

Note that the sign for ${\mathrm{\Psi }}_{\cal W}\left( {\sigma _l^z \leftrightarrow \sigma _m^z} \right){\mathrm{sinh}}\left( {2J_{lm}^{xy}\delta _\tau } \right)$ term is different from that in Eq. (39).

A solution of Eq. (65) is obtained as

66 $${\mathrm{\Delta }}W_{lj} = - {\mathrm{\Delta }}W_{mj} = - \frac{1}{2}\left( {W_{lj} - W_{mj}} \right),$$

where *W*_*nj*_ (*n* = *l*, *m*) is updated to $\bar W_{nj}$ with the increment Δ*W*_*nj*_ as $\bar W_{nj}$ = $W_{nj} + {\mathrm{\Delta }}W_{nj}$. The new couplings $W_{j[l]}^\prime$, *Z*_*lmj*_ and $W_{n[l]}^{\prime\prime}$ are also given by

67 $$W_{j[l]}^\prime = - Z_{lmj} = - \frac{1}{2}\left( {W_{lj} - W_{mj}} \right)$$

and

68 $$\begin{array}{c}W_{l[l]}^{\prime\prime} = \frac{1}{4}\left[ {{\mathrm{log}}\left[ { - {\mathrm {e}}^{ - 2a_{l - m}}{\mathrm{tanh}}\left( {2J_{lm}^{xy}\delta _\tau } \right)} \right]} \right.\\ \left. { + 2{\mathrm{arcosh}}\left[ {\frac{{{\mathrm {e}}^{ - 2J_{lm}^z\delta _\tau }}}{{\sqrt { - 2{\mathrm {e}}^{ - 2a_{l - m}}{\mathrm{sinh}}\left( {4J_{lm}^{xy}\delta _\tau } \right)} }}} \right]} \right]\end{array}$$

69 $$\begin{array}{c}W_{m[l]}^{\prime\prime} = \frac{1}{4}\left[ { - {\mathrm{log}}\left[ { - {\mathrm {e}}^{ - 2a_{l - m}}{\mathrm{tanh}}\left( {2J_{lm}^{xy}\delta _\tau } \right)} \right]} \right.\\ \left. { + {\mathrm{2arcosh}}\left[ {\frac{{{\mathrm {e}}^{ - 2J_{lm}^z\delta _\tau }}}{{\sqrt { - 2{\mathrm {e}}^{ - 2a_{l - m}}{\mathrm{sinh}}\left( {4J_{lm}^{xy}\delta _\tau } \right)} }}} \right]} \right]\end{array}$$

with *a*_*l*−*m*_ = *a*_*l*_ − *a*_*m*_. On a bipartite lattice, to avoid the negative sign (or complex phase) problem we need to keep $W_{l[l]}^{\prime\prime}$ and $W_{m[l]}^{\prime\prime}$ real. This can be achieved by choosing *a*_*l*_ = 0 for any *l* if *J*_*lm*_ < 0 (ferromagnetic case). For *J*_*lm*_ > 0 (antiferromagnetic case), *a*_*l*_ = *nπi* with an arbitrary integer *n* if the site *l* belongs to the sub-lattice A and *a*_*l*_ = (*n* + 1/2)*πi* if *l* belongs to the sub-lattice B. This local gauge for *J*_*lm*_ > 0 is equivalent to the transformation $J_{lm}^{xy} \to - J_{lm}^{xy}$ and *a*_*l*_ = 0 for any site *l*. We further notice that $W_{m[l]}^{\prime\prime}$ can be taken positive if we take a sufficiently small *δ*_*τ*_ in Eq (69), with the leading order term $- {\mathrm{log}}\left( {2J_{lm}^{xy}\delta _\tau } \right){\mathrm{/}}2$. On the other hand, in Eq. (68), the leading order term is negative (=−*J*_*lm*_*δ*_*τ*_).

To recover the original form of the DBM, we first use Eq. (21) with the replacement $s_1 \to \sigma _n^z$, *s*_2_ → *d*_[*l*]_, *s*_3_ → *h*_[*n*]_, *C* → *D*_*n*_, $V \to W_{n[l]}^{\prime\prime}$ $\tilde V_1 \to W_{n[n]}$, and $\tilde V_2 \to W_{[n][l]}^\prime$ for *n* = *l*, *m*. Then a solution for *D*_*n*_, *W*_*n*[*n*]_, and $W_{[n][l]}^\prime$ are represented by using $W_{n[l]}^{\prime\prime}$ as

70 $$D_n = \frac{1}{2}{\mathrm{exp}}\left[ { - W_{n[l]}^{\prime\prime} } \right]$$

71 $$W_{n[n]} = W_{[n][l]}^\prime = \frac{1}{2}{\mathrm{arcosh}}\left( {{\mathrm{exp}}\left[ {2W_{n[l]}^{\prime\prime} } \right]} \right),$$

for positive $W_{n[l]}^{\prime\prime}$ and

72 $$D_n = \frac{1}{2}{\mathrm{exp}}\left[ {W_{n[l]}^{\prime\prime} } \right]$$

73 $$W_{n[n]} = - W_{[n][l]}^\prime = \frac{1}{2}{\mathrm{arcosh}}\left( {{\mathrm{exp}}\left[ { - 2W_{n[l]}^{\prime\prime} } \right]} \right),$$

for negative $W_{n[l]}^{\prime\prime}$ to give real *W*_*n*[*n*]_ and $W_{[n][l]}^\prime$.

To completely recover the original DBM form, we next use Eq. (26) by replacing *σ*_1_ with $\sigma _l^z$, *σ*_2_ with $\sigma _m^z$, *d*_1_ with *d*_[*l*]_, *d*_2_ with *d*_[*lm*]_, *h*_1_ with *h*_*j*_, *h*_2_ with *h*_[*lm*1]_, *h*_3_ with *h*_[*lm*2]_, and *V* with *Z*_*lmj*_.

With these solutions, by ignoring the trivial constant factors including *D*_*l*_ and *D*_*m*_, the evolution is described by introducing two deep and four hidden additional variables *d*_[*l*]_, *d*_[*lm*]_, *h*_[*l*]_, *h*_[*m*]_, *h*_[*lm*1]_, and *h*_[*lm*2]_ as

74 $$\begin{array}{c}{\mathrm{\Psi }}_{\bar {\cal W}}\left( {\sigma ^z} \right) = \mathop {\sum}\limits_{\{ \bar h,\bar d\} } {\kern 1pt} P_1\left( {\sigma ^z,h} \right)P_2(h,d){\mathrm{exp}}\left[ {\mathop {\sum}\limits_{j,n = l,m} {\kern 1pt} \sigma _n^zh_j{\mathrm{\Delta }}W_{nj}} \right.\\ + \mathop {\sum}\limits_j h_jd_{[l]}W_{j[l]}^\prime + \mathop {\sum}\limits_{n = l,m} {\kern 1pt} h_{[n]}\left( {\sigma _n^zW_{n[n]} + d_{[l]}W_{[n][l]}^\prime } \right)\\ \left. { + d_{[lm]}\mathop {\sum}\limits_j {\kern 1pt} h_jZ_{lmj} + \frac{{i\pi }}{4}\left( {h_{[lm1]} + h_{[lm2]}} \right)\left( {\sigma _l^z + \sigma _m^z + d_{[l]} + d_{[lm]}} \right)} \right],\end{array}$$

where $\left\{ {\bar h,\bar d} \right\}$ is a set consisting of the existing and new neurons.

### Code availability

Computer codes to create the deep Boltzmann machine networks for each model are provided as Supplementary Software 1–4. Other code written for and used in this study is available from the corresponding author upon reasonable request.

## Electronic supplementary material

Supplementary Information

Description of Additional Supplementary Files

Supplementary Software 1

Supplementary Software 2

Supplementary Software 3

Supplementary Software 4

## Data Availability

The datasets generated during and/or analyzed during the current study are available from the corresponding author on reasonable request.
