# Supplementary material for: Constructing exact representations of quantum many-body systems with deep neural networks
Source: Nat Commun. 2018 Dec 14;9:5322. doi: 10.1038/s41467-018-07520-3 (PMC6294148; doi:10.1038/s41467-018-07520-3)
Supplement: Supplementary file 1 — Supplementary Information [file 41467_2018_7520_MOESM1_ESM.pdf]

Constructing exact representations of quantum many-body systems with deep neural networks
–Supplementary Information–

Giuseppe Carleo
Center for Computational Quantum Physics, Flatiron Institute, 162 5th Avenue, New York, NY 10010, USA and Institute for Theoretical Physics, ETH Zurich, Wolfgang-Pauli-Str. 27, 8093 Zurich, Switzerland

Yusuke Nomura and Masatoshi Imada
Department of Applied Physics, The University of Tokyo, 7-3-1 Hongo, Bunkyo-ku, Tokyo 113-8656, Japan

Supplementary Notes

Supplementary Note 1: Deep Boltzmann Machines

Deep Boltzmann machine representation of quantum states. In the main text we have considered a representation of the many-body wave-function in terms of a two-layers deep Boltzmann Machine (DBM). In the following we specialize to the case of N spin 1/2 particles, described by the quantum numbers $|\sigma^z\rangle = |\sigma^z_1 \dots \sigma^z_N\rangle$ with $\sigma^z_i = \pm 1$. Then, we represent the amplitudes $\langle \sigma^z_1 \dots \sigma^z_N | \Psi \rangle \equiv \Psi(\sigma^z)$ in the DBM form:

$$\Psi_{\mathcal{W}}(\sigma^z) = \sum_{\{h\}} e^{\sum_i a_i \sigma^z_i} e^{\sum_{ij} \sigma^z_i h_j W_{ij} + \sum_j b_j h_j} \sum_{\{d\}} e^{\sum_{jk} h_j d_k W'_{jk} + \sum_k b'_k d_k}. \tag{1}$$

Here, we have introduced M hidden units h_j, M' deep units d_k, and a set of couplings and bias terms $\mathcal{W} \equiv (a, b, b', W, W')$. Hereafter, we call the neurons in the 1st hidden layer just hidden neurons and distinguish them from the neurons in the 2nd hidden layer, which are called deep neurons.

All those parameters, in general, must be taken complex-valued to represent a generic many-body state. The hidden and deep units are taken here to be of spin 1/2, i.e. $h_j = \pm 1$, $d_k = \pm 1$, and the summations are over all the possible values of those variables. From a pictorial point of view, the DBM architecture features direct connections (interactions) between nearest-neighboring layers. In particular, the visible layer of physical degrees of freedom $(\sigma^z_1 \dots \sigma^z_N)$ is connected only to the first layer of hidden variables $(h_1 \dots h_M)$, whereas the first layer is connected both to the visible spins and to the deep spins $(d_1 \dots d_{M'})$.

For the following derivations, it is useful to write the DBM amplitudes as:

$$\Psi_{\mathcal{W}}(\sigma^z) = \sum_{\{h,d\}} P_1(\sigma^z, h) P_2(h, d), \tag{2}$$

where we have introduced the two quantities:

$$P_1(\sigma^z, h) = e^{\sum_i a_i \sigma^z_i} e^{\sum_{ij} \sigma^z_i h_j W_{ij} + \sum_j b_j h_j} \tag{3}$$

$$P_2(h, d) = e^{\sum_{jk} h_j d_k W'_{jk} + \sum_k b'_k d_k}. \tag{4}$$

Notice that, in general, those weights are complex-valued, and cannot be interpreted as genuine Boltzmann weights. From these expressions, it is also straightforward to see that the Restricted Boltzmann Machine (RBM) expression for the wave-function is recovered when $M' = 0$, i.e. taking

$$\Psi^{\text{RBM}}_{\mathcal{W}}(\sigma^z) = \sum_{\{h\}} P_1(\sigma^z, h) \tag{5}$$

$$= e^{\sum_i a_i \sigma^z_i} \Pi^M_j 2\cosh\left(\sum^N_i \sigma^z_i h_j W_{ij} + b_j \right), \tag{6}$$

where we have explicitly performed the summation of the hidden variables. At variance with the RBM case, in the more general case when $M' > 0$, it is not possible to analytically obtain the DBM amplitudes.

Useful gadgets in constructing DBM neural network. In the Methods we have discussed several useful identities to decompose spin interactions. In particular, those identities are very useful if we need more complicated interactions between the visible spins σ^z, hidden variables h and deep variables d beyond the standard form Eq. (1). For the sake of completeness of this Supplementary Information, we reproduce here the identities for decomposing two-body, three-body, and four-body interactions.

The first identity reads

$$e^{s_1 s_2 V} = C \sum_{s_3 = \pm 1} e^{s_1 s_3 \tilde{V}_1 + s_2 s_3 \tilde{V}_2} = 2C \cosh(s_1 \tilde{V}_1 + s_2 \tilde{V}_2). \tag{7}$$

with

$$C = \frac{1}{2} e^{-|V|} \tag{8}$$

$$\tilde{V}_1 = \frac{1}{2} \text{arcosh}(e^{2|V|}) \tag{9}$$

$$\tilde{V}_2 = \text{sgn}(V) \times \tilde{V}_1 \tag{10}$$

for Ising variables s_1 and s_2, and a real interaction V. This is the gadget for decomposing two-body interactions discussed in Methods. In the following, we will use this identity to decompose either interactions between visible (physical spins) (in that case s_1 and s_2 are both σ^z variables), or to decompose direct interactions between a σ^z spin and a deep unit d.

Another identity (decomposition of four-body interaction) is

$$\begin{aligned} e^{s_1 s_2 s_3 s_4 V} &= \frac{1}{4} \sum_{s_5, s_6, s_7} \exp\left[i\frac{\pi}{4}(s_5+s_6)(s_1+s_2+s_3+s_7)\right] \exp(s_4 s_7 V) \\ &= \sum_{s_7} \cos^2\left[\frac{\pi}{4}(s_1+s_2+s_3+s_7)\right] \exp(s_4 s_7 V) \end{aligned} \tag{11}$$

for Ising variables s_i with $i = 1, \cdots, 4$. Although we have introduced complex couplings in the first line, each term in the summation in the second line of Eq. (11) is positive definite if V is real. The second line remains nonzero only for $s_7 = 1$ if $s_1 s_2 s_3 = 1$ and only for $s_7 = -1$ if $s_1 s_2 s_3 = -1$, which proves the identity. This identity with s_1 and s_2 as physical variables, s_4, s_5, and s_6 as hidden variables, and s_3 and s_7 as deep variables, which reads

$$e^{\sigma_1 \sigma_2 d_1 h_1 V} = \frac{1}{4} \sum_{h_2, h_3, d_2} \exp\left[i\frac{\pi}{4}(h_2+h_3)(\sigma_1+\sigma_2+d_1+d_2)\right] \exp(h_1 d_2 V), \tag{12}$$

will be used in Sec. 2 B 3. Note that the right hand side fits the DBM structure.

Although identities for decomposing three-body interactions are not used in the following derivation, it is nonetheless useful to show them:

$$\begin{aligned} e^{s_1 s_2 s_3 V} &= \frac{1}{4} \sum_{s_4, s_5, s_6} \exp\left[i\frac{\pi}{4}(s_4+s_5)(s_1+s_2+s_3+s_6)\right] \exp(s_6 V) \\ &= \sum_{s_6} \cos^2\left[\frac{\pi}{4}(s_1+s_2+s_3+s_6)\right] \exp(s_6 V). \end{aligned} \tag{13}$$

This gadget for three-body interactions is obtained by fixing $s_4 = 1$ in Eq. (11) (and changing variables). Alternative form is obtained by replacing s_3 with 1 in Eq. (11), which gives,

$$\begin{aligned} e^{s_1 s_2 s_3 V} &= \frac{1}{4} \sum_{s_4, s_5, s_6} \exp\left[i\frac{\pi}{4}(s_4+s_5)(s_1+s_2+s_6+1)\right] \exp(s_3 s_6 V) \\ &= \sum_{s_6} \cos^2\left[\frac{\pi}{4}(s_1+s_2+s_6+1)\right] \exp(s_3 s_6 V). \end{aligned} \tag{14}$$

As we see, the gadgets for three-body interactions [Eqs. (13) and (14)] have been derived from the gadget for four-body interactions [Eq. (11)] trivially.

Gadgets for two-body interactions which are different from Eq. (7) can also be obtained from Eq. (11) by fixing two variables out of s_1, s_2, s_3, s_4 to be 1. These could be used instead of (7), although we employ (7) in the formalism below for the decoupling of the two-body interaction.

Supplementary Note 2: Representing Ground-States

As discussed in the main text, our goal is to construct explicit DBM representations of ground-states of local Hamiltonians. This goal is achieved by finding a representation of the imaginary-time evolved state:

$$|\Psi(\tau)\rangle = e^{-\tau\mathcal{H}}|\Psi_0\rangle, \tag{15}$$

where $|\Psi_0\rangle$ is empty RBM ($\langle\sigma^z|\Psi_0\rangle$ = const.) or pre-optimized RBM state, converging to the exact ground-state for large enough τ. To achieve this goal we first consider a second-order Trotter-Suzuki decomposition:

$$|\Psi(\tau)\rangle = \mathcal{G}_1(\delta_\tau/2)\mathcal{G}_2(\delta_\tau)\ldots\mathcal{G}_1(\delta_\tau)\mathcal{G}_2(\delta_\tau)\mathcal{G}_1(\delta_\tau/2)|\Psi_0\rangle, \tag{16}$$

where δ_τ is a small time step, the Hamiltonian is decomposed into two non-commuting parts, $\mathcal{H} = \mathcal{H}_1 + \mathcal{H}_2$, and $\mathcal{G}_v(\delta_\tau) = e^{-\mathcal{H}_v \delta_\tau}$ are short-time propagators. For given Hamiltonian, we then need to find specific rules to apply the short-time propagators to a generic DBM, and obtain a new (time-evolved) DBM, possibly with a larger total number of hidden and deep neurons. In the following, we show concrete examples for the transverse-field Ising and Heisenberg models.

A. Transverse-Field Ising model

Let us start with the case of the transverse-field Ising model. We consider a Trotter-Suzuki decomposition of the imaginary-time propagator, into two parts: $\mathcal{H}_1 = -\sum_i \Gamma_i \sigma_i^x$, and $\mathcal{H}_2 = \sum_{l<m} V_{lm}\sigma_l^z\sigma_m^z$. In the following derivation, we assume that Γ_i is positive ($\Gamma_i > 0$). In this case, we look for a solution with zero bias terms: $a_i = b_j = b'_k = 0, \forall i, j, k$. The case of negative Γ_i can also be treated, and is discussed more in detail at the end of this section.

Interaction propagator. The interaction propagator $e^{-\delta_\tau V_{lm}\sigma_l^z\sigma_m^z}$ is diagonal in the σ^z basis, and applying it to a DBM will lead to a modification in the DBM parameters. In particular, the goal is to satisfy the equation:

$$\langle\sigma^z|e^{-\delta_\tau V_{lm}\sigma_l^z\sigma_m^z}|\Psi_{\mathcal{W}}\rangle = C\langle\sigma^z|\Psi_{\bar{\mathcal{W}}}\rangle, \tag{17}$$

i.e. to explicitly find a set of parameters $\bar{\mathcal{W}}$ that satisfies the previous equation for all the possible $\langle\sigma^z|$, and for an arbitrary constant C.

We can achieve this goal adding a hidden unit in the first layer, $h_{[lm]}$ such that it is only connected to the visible spins: $W'_{[lm]k} = 0, \forall k$. The new wave function has then the form:

$$\Psi_{\bar{\mathcal{W}}}(\sigma^z) = \sum_{\{h,d\}}\sum_{h_{[lm]}} P_1(\sigma^z,h)P_2(h,d)e^{\sigma_l^z W_{l[lm]}h_{[lm]}+\sigma_m^z W_{m[lm]}h_{[lm]}} \tag{18}$$

$$= 2\cosh\left(\sigma_l^z W_{l[lm]} + \sigma_m^z W_{m[lm]}\right)\Psi_{\mathcal{W}}(\sigma^z). \tag{19}$$

Equation (17) is then satisfied if

$$e^{-\delta_\tau V_{lm}\sigma_l^z\sigma_m^z} = 2C\cosh\left(\sigma_l^z W_{l[lm]} + \sigma_m^z W_{m[lm]}\right) \tag{20}$$

for all the possible values of σ_l^z and σ_m^z. By using the gadget Eq. (7), the new parameters $W_{l[lm]}$ and $W_{m[lm]}$ are given by

$$W_{l[lm]} = \frac{1}{2}\text{arcosh}\left(e^{2|V_{lm}|\delta_\tau}\right) \tag{21}$$

$$W_{m[lm]} = -\text{sgn}(V_{lm}) \times W_{l[lm]}. \tag{22}$$

Transverse-field propagator. The propagator involving the transverse-field $e^{\delta_\tau\Gamma_l\sigma_l^x}$ is off-diagonal in σ^z basis. For this off-diagonal part, we must solve a slightly more involved equation:

$$\langle\sigma^z|e^{\delta_\tau\Gamma_l\sigma_l^x}|\Psi_{\mathcal{W}}\rangle = \Psi_{\mathcal{W}}(\sigma^z)\times\cosh(\Gamma_l\delta_\tau) + \Psi_{\mathcal{W}}(\sigma_1^z,\cdots-\sigma_l^z,\ldots,\sigma_N^z)\times\sinh(\Gamma_l\delta_\tau) \tag{23}$$

$$= C\langle\sigma^z|\Psi_{\bar{\mathcal{W}}}\rangle, \tag{24}$$

for the new parameters $\bar{\mathcal{W}}$, and for an arbitrary finite normalization constant C. In turn, this equation is equivalent to:

$$\sum_{\{h,d\}} P_1(\sigma^z,h)P_2(h,d)\left[1+\tanh(\Gamma_l\delta_\tau)e^{-2\sigma_l^z\sum_j h_j W_{lj}}\right] = C\Psi_{\bar{\mathcal{W}}}(\sigma^z). \tag{25}$$

We look for a solution by adding one deep neuron $d_{[l]}$ and creating new couplings $W'_{j[l]}$ to the existing hidden neurons h_j which are connected to σ_l^z. We also allow for changes in the existing interaction parameters. In particular we set the new couplings to be $\bar{W}_{lj} = W_{lj} + \Delta W_{lj}$, (with ΔW_{lj} to be determined).

Moreover, we introduce one hidden neuron $h_{[l]}$ coupled to σ_l^z and $d_{[l]}$ through the interactions $W_{l[l]}$ and $W'_{[l][l]}$, respectively. If we trace out $h_{[l]}$, the hidden neuron $h_{[l]}$ mediates the interaction between σ_l^z and $d_{[l]}$ (denoted as $W''_{l[l]}$).

With this choice, we have (in the representation where $h_{[l]}$ is traced out):

$$\Psi_{\bar{\mathcal{W}}}(\sigma^z) = \sum_{\{h,d\}}\sum_{d_{[l]}} P_1(\sigma^z,h)P_2(h,d)e^{\sigma_l^z\sum_j\Delta W_{lj}h_j + d_{[l]}\sum_j h_j W'_{j[l]} + \sigma_l^z d_{[l]}W''_{l[l]}}. \tag{26}$$

The equations to be verified are obtained considering the two possible values of $\sigma_l^z = \pm 1$:

$$e^{\sum_j h_j\left(\Delta W_{lj}+W'_{j[l]}\right)+W''_{l[l]}} + e^{\sum_j h_j\left(\Delta W_{lj}-W'_{j[l]}\right)-W''_{l[l]}} = C\times\left(1+\tanh(\Gamma_l\delta_\tau)e^{-2\sum_j h_j W_{lj}}\right) \tag{27}$$

$$e^{\sum_j h_j\left(-\Delta W_{lj}+W'_{j[l]}\right)-W''_{l[l]}} + e^{\sum_j h_j\left(-\Delta W_{lj}-W'_{j[l]}\right)+W''_{l[l]}} = C\times\left(1+\tanh(\Gamma_l\delta_\tau)e^{2\sum_j h_j W_{lj}}\right). \tag{28}$$

This equation has a solution if the hidden unit interactions on the l.h.s. and on the r.h.s match, i.e. when:

$$\Delta W_{lj} + W'_{j[l]} = -2W_{lj} \tag{29}$$

$$\Delta W_{lj} - W'_{j[l]} = 0, \tag{30}$$

which in turn are verified when

$$W'_{j[l]} = -W_{lj} \tag{31}$$

$$\Delta W_{lj} = -W_{lj}, \tag{32}$$

and if

$$W''_{l[l]} = \frac{\log\tanh(\Gamma_l \delta_\tau)}{2}. \tag{33}$$

When $\Gamma_l > 0$, $W''_{l[l]}$ is real. By using Eq. (7) with the following replacement $s_1 \to \sigma^z_l$, $s_2 \to d_{[l]}$, $s_3 \to h_{[l]}$, $V \to W''_{l[l]}$, $\tilde{V}_1 \to W_{l[l]}$ and $\tilde{V}_2 \to W'_{[l][l]}$, the last condition determines the real couplings $W_{l[l]}$ and $W'_{[l][l]}$, which read

$$W_{l[l]} = \frac{1}{2}\text{arcosh}\left(\frac{1}{\tanh(\Gamma_l \delta_\tau)}\right) \tag{34}$$

$$W'_{[l][l]} = -W_{l[l]}. \tag{35}$$

Notice that because of condition (32), after applying the off-diagonal propagator all the interactions W_{lj} between spin l and hidden units h_j are set to zero. However, because of condition (34), the spin l is reconnected to the new hidden unit $h_{[l]}$ with the $W_{l[l]}$ interaction.

Negative transverse field. When $\Gamma_i < 0$, it is still possible to recover a DBM representation with purely real interaction weights W and W'. In order to do so, we apply the gauge transformation $\sigma^x_i \to -\sigma^x_i$ and $\sigma^y_i \to -\sigma^y_i$ (π spin rotation around the z axis), which maps onto the Hamiltonian with positive Γ_i. This gauge transformation can be achieved by taking a finite bias terms a_i in Eq. (1) as $a_i = i\pi/2$ and fix them during the imaginary time evolution. With this complex bias term $a_i = i\pi/2$, $|\uparrow\rangle$ ($|\downarrow\rangle$) state at the ith site acquires a phase as follows $|\uparrow\rangle \to e^{i\frac{\pi}{2}}|\uparrow\rangle = i|\uparrow\rangle$ ($|\downarrow\rangle \to e^{-i\frac{\pi}{2}}|\downarrow\rangle = -i|\downarrow\rangle$), which is equivalent to a π spin rotation around the z axis. In the case when Γ_i is originally positive, we can set all the bias terms $\{a, b, b'\}$ to be zero.

B. Heisenberg Model

We now consider the case of the Heisenberg model, whose Hamiltonian reads

$$\mathcal{H} = \sum_{\langle lm \rangle} \mathcal{H}_{lm} \tag{36}$$

$$\mathcal{H}_{lm} = \mathcal{H}^z_{lm} + \mathcal{H}^{xy}_{lm} \tag{37}$$

$$\mathcal{H}^z_{lm} = J^z_{lm}\sigma^z_l\sigma^z_m \tag{38}$$

$$\mathcal{H}^{xy}_{lm} = J^{xy}_{lm}(\sigma^x_l\sigma^x_m + \sigma^y_l\sigma^y_m) = 2J^{xy}_{lm}(\sigma^+_l\sigma^-_m + \sigma^-_l\sigma^+_m) \tag{39}$$

with $J^z_{lm} = J^{xy}_{lm} = J$. We write the Hamiltonian in a general form because the following DBM algorithm can be straightforwardly extended to the more general case of anisotropic/disordered bonds. As a starting point for our construction, we decompose the Hamiltonian into pieces by a Trotter-Suzuki decomposition of the imaginary-time propagator: $e^{-\delta_\tau \mathcal{H}} \sim \prod_{\langle lm \rangle} e^{-\delta_\tau \mathcal{H}_{lm}} + O(\delta_\tau{}^2)$. Then in this Section, we represent $e^{-\delta_\tau \mathcal{H}_{lm}}$ by using the DBM in three different forms, which are all exact. By taking δ_τ small enough and operating $e^{-\delta_\tau \mathcal{H}_{lm}}$ many times, those constructions ensure that the ground state is obtained with any controlled accuracy.

For $e^{-\delta_\tau \mathcal{H}_{lm}}$, and the antiferromagnetic exchange $J^z_{lm}, J^{xy}_{lm} > 0$, if the lattice is bipartite, we further apply a local gauge transformation by π rotation around z axis in the spin space as

$$\sigma^x \to -\sigma^x \quad \text{and} \quad \sigma^y \to -\sigma^y \tag{40}$$

on one of the sublattices, which gives a $-$ sign for the $\sigma^x_l\sigma^x_m$ and $\sigma^y_l\sigma^y_m$ interactions. It is equivalent to the following transformation in the couplings:

$$J^{xy}_{lm} \to -J^{xy}_{lm}. \tag{41}$$

The gauge transformation enables to design a DBM neural network with real couplings $\{W, W'\}$ except for those necessary to enforce local constraints on the values of deep neuron spins (see more detail about the constraint in the following sections). Overall, we show in the following that the 3 different DBM constructions have no negative sign problem.

On the bipartite lattice, the Suzuki-Trotter decomposition is frequently expressed by decomposing the Hamiltonian $\mathcal{H}$ into several groups. For instance, on the one dimensional chain, if it is natural to decompose it into odd and even bonds:

$$\mathcal{H}_1 = \sum_{\langle l,m\rangle \in \text{odd bond}} \mathcal{H}_{lm}, \quad \mathcal{H}_2 = \sum_{\langle l,m\rangle \in \text{even bond}} \mathcal{H}_{lm}, \tag{42}$$

further decompositions $e^{-\delta_\tau \mathcal{H}_1} = \prod_{\langle l,m\rangle \in \text{ odd bond}} e^{-\delta_\tau \mathcal{H}_{lm}}$ and $e^{-\delta_\tau \mathcal{H}_2} = \prod_{\langle l,m\rangle \in \text{ even bond}} e^{-\delta_\tau \mathcal{H}_{lm}}$ contain commuting elements and are therefore exact. For the square lattice, a similar procedure requires the decomposition of the Hamiltonian into 4 parts, in a checkerboard fashion. In all cases, the fundamental ingredient to represent the ground-state as a DBM is to find an exact expression for the bond propagator, $e^{-\delta_\tau \mathcal{H}_{lm}}$, when applied to an existing DBM state.

In the case of antiferromagnetic Heisenberg model after the gauge transformation on the bipartite lattice, we must solve, for each bond,

$$\begin{aligned}
&\langle \sigma^z | e^{\delta_\tau J^{xy}_{lm}\left(\sigma^x_l \sigma^x_m + \sigma^y_l \sigma^y_m\right) - \delta_\tau J^z_{lm} \sigma^z_l \sigma^z_m} | \Psi_{\mathcal{W}} \rangle \\
&\quad = \delta_{\sigma^z_l, \sigma^z_m} e^{-\delta_\tau J^z_{lm}} \Psi_{\mathcal{W}}(\sigma^z) + (1 - \delta_{\sigma^z_l, \sigma^z_m}) e^{\delta_\tau J^z_{lm}} \left(\Psi_{\mathcal{W}}(\sigma^z) \times \cosh(2 J^{xy}_{lm} \delta_\tau) + \Psi_{\mathcal{W}}(\sigma^z_l \leftrightarrow \sigma^z_m) \times \sinh(2 J^{xy}_{lm} \delta_\tau) \right) \\
&\quad = C \langle \sigma^z | \Psi_{\bar{\mathcal{W}}} \rangle.
\end{aligned} \tag{43}$$

It is also useful to explicitly write the expression for the exchange term in the second line above:

$$\begin{aligned}
&\Psi_{\mathcal{W}}(\sigma^z) \times \cosh(2 J^{xy}_{lm} \delta_\tau) + \Psi_{\mathcal{W}}(\sigma^z_l \leftrightarrow \sigma^z_m) \times \sinh(2 J^{xy}_{lm} \delta_\tau) \\
&\quad = \sum_{\{h,d\}} P_1(\sigma^z, h) P_2(h, d) \left[\cosh(2 J^{xy}_{lm} \delta_\tau) + \sinh(2 J^{xy}_{lm} \delta_\tau) e^{(\sigma^z_m - \sigma^z_l) \sum_j h_j \left(W_{lj} - W_{mj}\right)} \right].
\end{aligned} \tag{44}$$

In the following derivations, for the antiferromagnetic Hamilonian ($J^z_{lm}, J^{xy}_{lm} > 0$) after the gauge transformation, we look for a solution with zero bias terms ($a_i,\ b_j,\ b'_k = 0, \forall i, j, k$). We can also derive a sign-problem free solution for the imaginary time evolution in the absence of the explicit gauge transformation by introducing complex bias term a_i. Indeed, in the "*2 deep, 4 hidden*" representation in Sec. 2 B 3, we will explicitly show that taking a specific set of complex bias term a_i on physical spins is equivalent to the gauge transformation, making a solution free from the sign problem.

1. 1 deep, 3 hidden *(1d-3h) representation*

Strategy. The first representation we propose is obtained adding one deep neuron $d_{[lm]}$, which gives new couplings $W'_{j[lm]}$ to the hidden units h_j connected to σ^z_l and σ^z_m. We also allow for changes in the existing DBM parameters. In particular we set the new couplings to be $\bar{W}_{lj} = W_{lj} + \Delta W_{lj}$, (with ΔW_{lj} to be determined). We introduce a coupling $W''_{l[lm]}$ between σ^z_l and $d_{[lm]}$, and a coupling $V_{[lm]}$ between σ^z_l and σ^z_m, which are both not allowed in the DBM architecture. By using the gadget Eq. (7), these interactions can be mediated by hidden neurons $h_{[lm1]}$ and $h_{[lm2]}$, respectively, and the DBM form is recovered. Furthermore, we look for a solution with a constraint: $d_{[lm]} = \sigma^z_l$ when $\sigma^z_l = \sigma^z_m$ (when $\sigma^z_l \neq \sigma^z_m$, the $d_{[lm]}$ value is not constrained). Imposing the constraint on the value of the deep unit is a crucial difference from the DBM solution for the TFI model. We will show that this constraint can be achieved by adding additional hidden neuron $h_{[lm3]}$ and introducing complex couplings ("$i\pi/6$" trick). We discuss this trick in more detail later.

In total, we introduce one deep and three hidden neurons. After tracing out the three hidden neurons $h_{[lm1]}$, $h_{[lm2]}$, and $h_{[lm3]}$, the new wave function reads

$$\Psi_{\bar{\mathcal{W}}}(\sigma^z) = \sum_{\{h,d\}} \sum_{\substack{d_{[lm]}=\pm 1 \\ d_{[lm]}=\sigma^z_l \text{ if } \sigma^z_l = \sigma^z_m}} P_1(\sigma^z, h) P_2(h, d) e^{\sigma^z_l \sum_j \Delta W_{lj} h_j + d_{[lm]} \sum_j h_j W'_{j[lm]} + d_{[lm]} \sigma^z_l W''_{l[lm]} + V_{[lm]} \sigma^z_l \sigma^z_m}. \tag{45}$$

Derivation for the update of parameters. The equations to be verified are then obtained considering all the possible values of $\sigma^z_l = \pm 1$ and $\sigma^z_m = \pm 1$, in addition to the constraints on $d_{[lm]}$ previously introduced. We then have two equations for $\sigma^z_l = \sigma^z_m = \pm 1$:

$$e^{\sum_j \left(\Delta W_{lj} + W'_{j[lm]}\right) h_j + W''_{l[lm]} + V_{[lm]}} = C \times \exp(-J^z_{lm} \delta_\tau) \tag{46}$$

$$e^{\sum_j \left(-\Delta W_{lj} - W'_{j[lm]}\right) h_j + W''_{l[lm]} + V_{[lm]}} = C \times \exp(-J^z_{lm} \delta_\tau), \tag{47}$$

and the other two equations for $\sigma^z_l = -\sigma^z_m = \pm 1$:

$$e^{\sum_j\left(\Delta W_{lj}+W'_{j[lm]}\right)h_j+W''_{l[lm]}-V_{[lm]}} + e^{\sum_j\left(\Delta W_{lj}-W'_{j[lm]}\right)h_j-W''_{l[lm]}-V_{[lm]}} \\ = C\times\exp(J^z_{lm}\delta_\tau)\left(\cosh(2J^{xy}_{lm}\delta_\tau)+\sinh(2J^{xy}_{lm}\delta_\tau)e^{-2\sum_j h_j(W_{lj}-W_{mj})}\right), \tag{48}$$

$$e^{\sum_j\left(-\Delta W_{lj}+W'_{j[lm]}\right)h_j-W''_{l[lm]}-V_{[lm]}} + e^{\sum_j\left(-\Delta W_{lj}-W'_{j[lm]}\right)h_j+W''_{l[lm]}-V_{[lm]}} \\ = C\times\exp(J^z_{lm}\delta_\tau)\left(\cosh(2J^{xy}_{lm}\delta_\tau)+\sinh(2J^{xy}_{lm}\delta_\tau)e^{2\sum_j h_j(W_{lj}-W_{mj})}\right). \tag{49}$$

These equations have a solution if the hidden unit interactions on the l.h.s. and on the r.h.s match, i.e. when:

$$\Delta W_{lj} + W'_{j[lm]} = 0 \tag{50}$$

$$\Delta W_{lj} - W'_{j[lm]} = -2(W_{lj} - W_{mj}) \tag{51}$$

which implies

$$\Delta W_{lj} = -W_{lj} + W_{mj} \tag{52}$$

$$W'_{j[lm]} = W_{lj} - W_{mj}. \tag{53}$$

Notice that the first condition gives $\bar{W}_{lj} = W_{lj} + \Delta W_{lj} = W_{mj}$, which is equivalent to cutting all connections from spin l to the hidden units and attaching the spin l to all the hidden units connected to spin m, with an interaction W_{mj}.

In order to match the coefficients we must also have:

$$W''_{l[lm]} + V_{[lm]} = \log C - J^z_{lm}\delta_\tau \tag{54}$$

$$W''_{l[lm]} - V_{[lm]} = \log C + \log\cosh(2J^{xy}_{lm}\delta_\tau) + J^z_{lm}\delta_\tau \tag{55}$$

$$-W''_{l[lm]} - V_{[lm]} = \log C + \log\sinh(2J^{xy}_{lm}\delta_\tau) + J^z_{lm}\delta_\tau, \tag{56}$$

which has the solution:

$$W''_{l[lm]} = -\left(\log\tanh(2J^{xy}_{lm}\delta_\tau)\right)/2 \tag{57}$$

$$V_{[lm]} = -\left(\log\cosh(2J^{xy}_{lm}\delta_\tau)\right)/2 - J^z_{lm}\delta_\tau \tag{58}$$

Recovery of standard DBM. The coupling $W''_{l[lm]}$ between the deep unit $d_{[lm]}$ and the visible spin σ^z_l is mediated by the hidden unit $h_{[lm1]}$ coupled to σ^z_l by $W_{l[lm1]}$ and $d_{[lm]}$ by $W'_{[lm1][lm]}$:

$$\exp(\sigma^z_l d_{[lm]} W''_{l[lm]}) = C_{[lm1]}\sum_{h_{[lm1]}}\exp(\sigma^z_l h_{[lm1]}W_{l[lm1]} + h_{[lm1]}d_{[lm]}W'_{[lm1][lm]}). \tag{59}$$

By using Eq. (7) with the following replacement $s_1 \to \sigma^z_l$, $s_2 \to d_{[lm]}$, $s_3 \to h_{[lm1]}$, $V \to W''_{l[lm]}$, $\tilde{V}_1 \to W_{l[lm1]}$ and $\tilde{V}_2 \to W'_{[lm1][lm]}$, $W_{l[lm1]}$ and $W'_{[lm1][lm]}$ are given by

$$W_{l[lm1]} = W'_{[lm1][lm]} = \frac{1}{2}\text{arcosh}\left(\frac{1}{\tanh(2J^{xy}_{lm}\delta_\tau)}\right). \tag{60}$$

Similarly, the coupling $V_{[lm]}$ between visible spins σ^z_l and σ^z_m is mediated by the hidden unit $h_{[lm2]}$ coupled to σ^z_l by $W_{l[lm2]}$ and σ^z_m by $W_{m[lm2]}$:

$$\exp(\sigma^z_l\sigma^z_m V_{[lm]}) = C_{[lm2]}\sum_{h_{[lm2]}}\exp(\sigma^z_l h_{[lm2]}W_{l[lm2]} + \sigma^z_m h_{[lm2]}W_{m[lm2]}). \tag{61}$$

By using Eq. (7) with the following replacement $s_1 \to \sigma^z_l$, $s_2 \to \sigma^z_m$, $s_3 \to h_{[lm2]}$, $V \to V_{lm}$, $\tilde{V}_1 \to W_{l[lm2]}$ and $\tilde{V}_2 \to W_{m[lm2]}$, $W_{l[lm2]}$ and $W_{m[lm2]}$ are given by

$$W_{l[lm2]} = -W_{m[lm2]} = \frac{1}{2}\text{arcosh}\left(\cosh(2J^{xy}_{lm}\delta_\tau)e^{2J^z_{lm}\delta_\tau}\right). \tag{62}$$

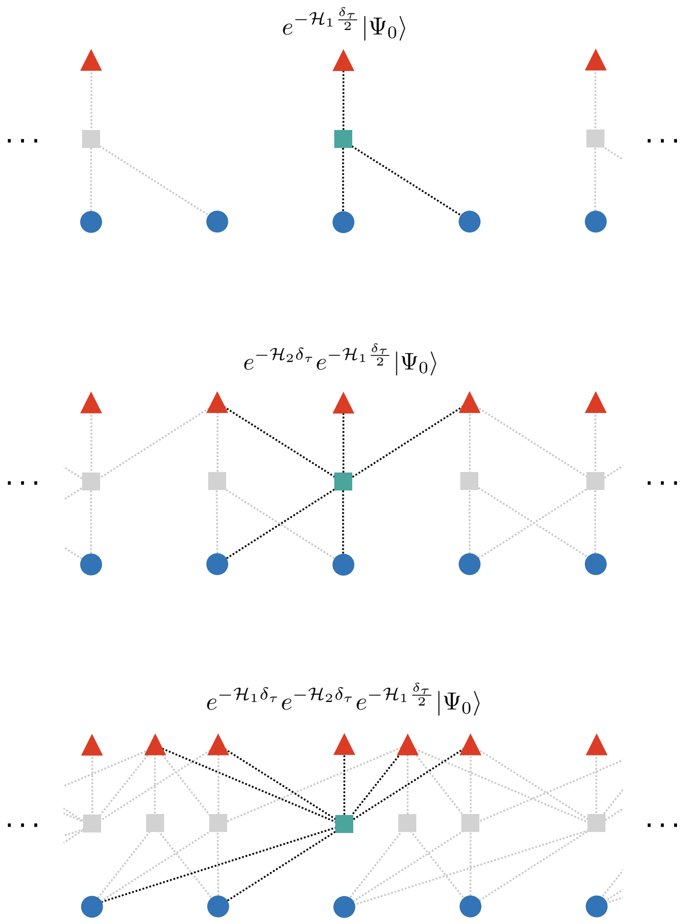

Supplementary Fig. 1. Imaginary-time evolution of complex couplings in 1d-3h construction for one-dimensional Heisenberg model. The figure shows how the complex couplings with weight $\pm i\pi/6$ evolve from an empty RBM ($\langle\sigma^z|\Psi_0\rangle$ = const.). Dots, squares, triangles indicate physical spins σ_i^z, hidden neurons h_j, and deep neurons d_k, respectively. For visibility, only hidden neurons having complex couplings and the associated complex couplings are shown. Therefore, at each imaginary-time evolution, one hidden neuron (called $h_{[lm3]}$ in the text) appears for each bond. One hidden neuron (green) and the associated couplings (black) are highlighted. As discussed in Step 2 in Fig. 3, at each evolution on σ_l^z and σ_m^z, the W couplings to σ_l^z are cut and σ_l^z is reconnected to the hidden neuron coupled to σ_m^z. By this "cut and reconnect" procedure, the positions of nonzero W couplings from a specific hidden neuron move, however, the W couplings stay local. On the other hand, the number of nonzero W' couplings increases by imaginary-time evolution, resulting in non-local structure of W' couplings. For the same reason, the real W couplings stay local, whereas the real W' couplings become nonlocal.

How to enforce the constraint $d_{[lm]} = \sigma_l^z$ when $\sigma_l^z = \sigma_m^z$ ("$i\pi/6$" trick). The constraint $d_{[lm]} = \sigma_l^z$ when $\sigma_l^z = \sigma_m^z$ can be exactly satisfied by introducing pure complex connections. We can replace the sum with the constraint in Eq. (45) as follows (we ignore trivial constant factor):

$$\sum_{\substack{d_{[lm]}=\pm 1 \\ d_{[lm]}=\sigma_l^z \text{ if } \sigma_l^z=\sigma_m^z}} \longrightarrow \sum_{d_{[lm]}} \sum_{h_{[lm3]}} e^{i\frac{\pi}{6}\left((\sigma_l^z+\sigma_m^z)h_{[lm3]}-h_{[lm3]}d_{[lm]}\right)} = \sum_{d_{[lm]}} 2\cos\left(\frac{\pi}{6}\left(\sigma_l^z+\sigma_m^z-d_{[lm]}\right)\right) \tag{63}$$

One can easily see that the cosine term in the rightmost part gives nonzero value only when $d_{[lm]} = \sigma_l^z$ if $\sigma_l^z = \sigma_m^z$. On the other hand, if $\sigma_l^z \neq \sigma_m^z$, both $d_{[lm]} \pm 1$ contributions survive.

Proof of no negative sign. Here, we show that the marginal probability density $\tilde{\Pi}'(\sigma^z, d, d') = \sum_{h,h'} \Pi(\sigma^z, h, h', d, d')$ obtained

by tracing out the hidden unit is non-negative definite. Therefore, we can perform the Metropolis sampling using $\tilde{\Pi}'$ density without suffering from the negative signs (see more detail on the sampling scheme in Sec. 3 B). To prove this, it is sufficient to show

$$\sum_{\{h\}} P_1(\sigma^z, h) P_2(h, d) \geq 0. \tag{64}$$

for all possible σ^z and d configurations.

In the 1d-3h representation, $i\pi/6$ complex couplings are originally introduced to put the constraint locally. However, as time evolves, these complex couplings become non-local (see Fig. 1). Because the pure complex couplings give cosine terms after tracing out hidden variables, they have a potential to give negative signs. Here, we prove that this is not the case.

We assume that Eq. (64) is satisfied for all possible σ^z and d after several steps of the imaginary time evolution. Then, we apply the bond propagator $e^{-\mathcal{H}_{lm}\delta_\tau}$ to obtain the new wave function. In the case when $\sigma^z_l = -\sigma^z_m = 1$, the solution in the 1d-3h representation can be rewritten as

$$\sum_{h_{[lm1]}, h_{[lm2]}, h_{[lm3]}} \bar{P}_1(\sigma^z, \bar{h}) \bar{P}_2(\bar{h}, d, d_{[lm]} = 1) = P_1(\sigma^z, h) P_2(h, d) \times (\text{positive constant}) \tag{65}$$

$$\sum_{h_{[lm1]}, h_{[lm2]}, h_{[lm3]}} \bar{P}_1(\sigma^z, \bar{h}) \bar{P}_2(\bar{h}, d, d_{[lm]} = -1) = P_1(\sigma^z_l \leftrightarrow \sigma^z_m, h) P_2(h, d) \times (\text{positive constant}) \tag{66}$$

where $\bar{P}_1 \times \bar{P}_2$ on the left hand side is the new weight after the imaginary time evolution, and $\{\bar{h}\}$ consists of the existing hidden neurons $\{h\}$ and the newly introduced hidden neurons $h_{[lm1]}$, $h_{[lm2]}$, and $h_{[lm3]}$. By taking the summation on the existing hidden variables on both sides, we get

$$\sum_{\{\bar{h}\}} \bar{P}_1(\sigma^z, \bar{h}) \bar{P}_2(\bar{h}, d, d_{[lm]} = 1) = \sum_{\{h\}} P_1(\sigma^z, h) P_2(h, d) \times (\text{positive constant}) \geq 0 \tag{67}$$

$$\sum_{\{\bar{h}\}} \bar{P}_1(\sigma^z, \bar{h}) \bar{P}_2(\bar{h}, d, d_{[lm]} = -1) = \sum_{\{h\}} P_1(\sigma^z_l \leftrightarrow \sigma^z_m, h) P_2(h, d) \times (\text{positive constant}) \geq 0 \tag{68}$$

Here, we used Eq. (64) to obtain the rightmost inequality. It proves that the new weight with the hidden variables being traced out is also non-negative. In the same way, we can show the non-negativeness of the new weight for $\sigma_l = -\sigma_m = -1$.

Next we consider the case $\sigma^z_l = \sigma^z_m = 1$. In this case,

$$\sum_{h_{[lm1]}, h_{[lm2]}, h_{[lm3]}} \bar{P}_1(\sigma^z, \bar{h}) \bar{P}_2(\bar{h}, d, d_{[lm]} = 1) = P_1(\sigma^z, h) P_2(h, d) \times (\text{positive constant}), \tag{69}$$

$$\sum_{h_{[lm1]}, h_{[lm2]}, h_{[lm3]}} \bar{P}_1(\sigma^z, \bar{h}) \bar{P}_2(\bar{h}, d, d_{[lm]} = -1) = 0. \tag{70}$$

By taking the summation on the existing hidden variables on both sides, we obtain

$$\sum_{\{\bar{h}\}} \bar{P}_1(\sigma^z, \bar{h}) \bar{P}_2(\bar{h}, d, d_{[lm]} = 1) = \sum_{h} P_1(\sigma^z, h) P_2(h, d) \times (\text{positive constant}) \geq 0 \tag{71}$$

$$\sum_{\{\bar{h}\}} \bar{P}_1(\sigma^z, \bar{h}) \bar{P}_2(\bar{h}, d, d_{[lm]} = -1) = 0. \tag{72}$$

Therefore, the non-negativeness of the weight is ensured. The proof for $\sigma^z_l = \sigma^z_m = -1$ case can be done in an analogous way.

We have proven that the new weight after applying the bond propagator $e^{-\mathcal{H}_{lm}\delta_\tau}$ is non negative for all the possible σ^z and $\bar{d}$ configurations:

$$\sum_{\{\bar{h}\}} \bar{P}_1(\sigma^z, \bar{h}) \bar{P}_2(\bar{h}, \bar{d}) \geq 0 \tag{73}$$

with $\{\bar{d}\}$ consisting of $\{d\}$ and $d_{[lm]}$. It ensures the non-negativeness of the weight at any time during the imaginary time evolution.

Summary of 1d-3h representation. The action the bond propagator is summarized as follows. First, the new deep neuron $d_{[lm]}$ is attached to the existing hidden neurons connected to σ^z_l and σ^z_m. Second, σ^z_l is disconnected to all hidden units and reconnected to the hidden units having finite couplings to σ^z_m ($\bar{W}_{lj} = W_{mj}$). Third, four couplings are inserted, involving new hidden neurons $h_{[lm1]}$ and $h_{[lm2]}$: $\sigma^z_l \leftrightarrow h_{[lm1]}$, $h_{[lm1]} \leftrightarrow d_{[l]}$, $\sigma^z_l \leftrightarrow h_{[lm2]}$ and $\sigma^z_m \leftrightarrow h_{[lm2]}$, Finally, the new hidden neuron $h_{[lm3]}$ puts the constraint on the $d_{[l]}$ sum by the imaginary couplings to σ^z_l, σ^z_m, and $d_{[l]}$.

By successively applying the imaginary-time evolutions, the W' couplings become nonlocal or long ranged. On the other hand, the W couplings stay local (see Fig. 1).

2. 2 deep, 6 hidden (2d-6h) representation

Strategy. We look for a solution where we add two deep neurons $d_{[l]}$ and $d_{[m]}$, giving new couplings $W'_{j[l]}$, $W'_{j[m]}$ to the existing hidden spins h_j connected to σ^z_l and σ^z_m. We also allow for changes in the existing W parameters: We set the new couplings to be $\bar{W}_{lj} = W_{lj} + \Delta W_{lj}$ and $\bar{W}_{mj} = W_{mj} + \Delta W_{mj}$ (with ΔW_{lj}, ΔW_{mj} to be determined). Furthermore, we add four hidden neurons $h_{[lm1]}$, $h_{[lm2]}$, $h_{[lm3]}$, and $h_{[lm4]}$ to mediate the interactions between $(\sigma^z_l, d_{[l]})$, $(\sigma^z_m, d_{[m]})$, $(\sigma^z_l, d_{[m]})$, and $(\sigma^z_m, d_{[l]})$, respectively. We solve the equation with the constraint $\sigma^z_l + \sigma^z_m = d_{[l]} + d_{[m]}$. This constraint can be achieved, for example, by adding two further hidden neurons ($h_{[lm5]}$ and $h_{[lm6]}$, respectively) and introducing complex connections ("$i\pi/4, i\pi/8$" trick). This trick will be discussed in detail later.

In total, we add two deep neurons ($d_{[l]}$ and $d_{[m]}$) and six hidden neurons ($h_{[lm1]}, \dots, h_{[lm6]}$). In the following, to make equations simple, we employ a representation in which the new hidden neurons are analytically traced out. The interactions between $(\sigma^z_l, d_{[l]})$, $(\sigma^z_m, d_{[m]})$, $(\sigma^z_l, d_{[m]})$, and $(\sigma^z_m, d_{[l]})$, which are mediated by 1st to 4th hidden neurons, will be denoted as $W''_{l[l]}$, $W''_{m[m]}$, $W''_{l[m]}$, and $W''_{m[l]}$, respectively. The 5th and 6th hidden neurons filter out $\sigma^z_l + \sigma^z_m \neq d_{[l]} + d_{[m]}$ contributions. With this setting, the new wave function is represented as

$$\Psi_{\bar{\mathcal{W}}}(\sigma^z) = \sum_{\{h,d\}} \sum_{\substack{d_{[l]},d_{[m]} \\ d_{[l]}+d_{[m]}=\sigma^z_l+\sigma^z_m}} P_1(\sigma^z, h) P_2(h, d)\, e^{\sum_j h_j (\Delta W_{lj}\sigma^z_l + W'_{j[l]} d_{[l]}) + \sum_j h_j (\Delta W_{mj}\sigma^z_m + W'_{j[m]} d_{[m]})} \times e^{\sigma^z_l (W''_{l[l]} d_{[l]} + W''_{l[m]} d_{[m]}) + \sigma^z_m (W''_{m[l]} d_{[l]} + W''_{m[m]} d_{[m]})}. \quad (74)$$

Derivation for the update of parameters. When the lth and mth physical spins are anti-parallel ($\sigma^z_l = -\sigma^z_m = \pm 1$), $d_{[l]} = -d_{[m]} = \pm 1$ contributions survive in the sum over $d_{[l]}$ and $d_{[m]}$ variables in Eq. (74), and thus the equations to be satisfied are

$$e^{\sum_j h_j \left(\Delta W_{l-m,j} + W'_{j[l]} - W'_{j[m]}\right) + W''_{l-m,[l]} - W''_{l-m,[m]}} + e^{\sum_j h_j \left(\Delta W_{l-m,j} - W'_{j[l]} + W'_{j[m]}\right) - W''_{l-m,[l]} + W''_{l-m,[m]}} = C e^{J^z_{lm}\delta_\tau} \left(\cosh(2J^{xy}_{lm}\delta_\tau) + \sinh(2J^{xy}_{lm}\delta_\tau) e^{-2\sum_j h_j W_{l-m,j}}\right) \quad (75)$$

for $\sigma^z_l = -\sigma^z_m = 1$ and

$$e^{\sum_j h_j \left(-\Delta W_{l-m,j} + W'_{j[l]} - W'_{j[m]}\right) - W''_{l-m,[l]} + W''_{l-m,[m]}} + e^{\sum_j h_j \left(-\Delta W_{l-m,j} - W'_{j[l]} + W'_{j[m]}\right) + W''_{l-m,[l]} - W''_{l-m,[m]}} = C e^{J^z_{lm}\delta_\tau} \left(\cosh(2J^{xy}_{lm}\delta_\tau) + \sinh(2J^{xy}_{lm}\delta_\tau) e^{2\sum_j h_j W_{l-m,j}}\right) \quad (76)$$

for $\sigma^z_l = -\sigma^z_m = -1$, respectively. Here, $W_{l-m,[\alpha]} = W_{l[\alpha]} - W_{m[\alpha]}$, $\Delta W_{l-m,[\alpha]} = \Delta W_{l[\alpha]} - \Delta W_{m[\alpha]}$, $W''_{l-m,[\alpha]} = W''_{l[\alpha]} - W''_{m[\alpha]}$ with $\alpha = l, m$.

When the lth and mth physical spins are parallel ($\sigma^z_l = \sigma^z_m = \pm 1$), only $d_{[l]} = d_{[m]} = \sigma^z_l = \sigma^z_m$ contribution survives in the sum over $d_{[l]}$ and $d_{[m]}$ variables in Eq. (74), and thus the equations to be satisfied are

$$e^{\sum_j h_j \left(\Delta W_{l+m,j} + W'_{j[l]} + W'_{j[m]}\right) + W''_{l+m,[l]} + W''_{l+m,[m]}} = C e^{-J^z_{lm}\delta_\tau} \quad (77)$$

for $\sigma^z_l = \sigma^z_m = 1$

$$e^{\sum_j h_j \left(-\Delta W_{l+m,j} - W'_{j[l]} - W'_{j[m]}\right) + W''_{l+m,[l]} + W''_{l+m,[m]}} = C e^{-J^z_{lm}\delta_\tau} \quad (78)$$

for $\sigma^z_l = \sigma^z_m = -1$, respectively. Here, $W_{l+m,[\alpha]} = W_{l[\alpha]} + W_{m[\alpha]}$, $\Delta W_{l+m,[\alpha]} = \Delta W_{l[\alpha]} + \Delta W_{m[\alpha]}$, $W''_{l+m,[\alpha]} = W''_{l[\alpha]} + W''_{m[\alpha]}$ with $\alpha = l, m$.

The equations (75), (76), (77), and (78) are satisfied if

$$\Delta W_{l-m,j} - W'_{j[l]} + W'_{j[m]} = -2W_{l-m,j}, \quad (79)$$

$$\Delta W_{l-m,j} + W'_{j[l]} - W'_{j[m]} = 0, \quad (80)$$

$$\Delta W_{l+m,j} + W'_{j[l]} + W'_{j[m]} = 0, \quad (81)$$

and

$$W''_{l-m,[l]} - W''_{l-m,[m]} = \log C + J^z_{lm}\delta_\tau + \log\cosh(2J^{xy}_{lm}\delta_\tau), \quad (82)$$

$$-W''_{l-m,[l]} + W''_{l-m,[m]} = \log C + J^z_{lm}\delta_\tau + \log\sinh(2J^{xy}_{lm}\delta_\tau), \quad (83)$$

$$W''_{l+m,[l]} + W''_{l+m,[m]} = \log C - J^z_{lm}\delta_\tau. \quad (84)$$

These conditions give

$$W'_{j[l]} = W_{lj}, \tag{85}$$
$$W'_{j[m]} = W_{mj}, \tag{86}$$
$$\Delta W_{lj} = -W_{lj}, \tag{87}$$
$$\Delta W_{mj} = -W_{mj}, \tag{88}$$

and

$$W''_{l[l]} = W''_{m[m]} = -\frac{J^z_{lm}\delta_\tau}{2} - \frac{1}{4}\log\sinh(2J^{xy}_{lm}\delta_\tau), \tag{89}$$
$$W''_{l[m]} = W''_{m[l]} = -\frac{J^z_{lm}\delta_\tau}{2} - \frac{1}{4}\log\cosh(2J^{xy}_{lm}\delta_\tau). \tag{90}$$

Recovery of standard DBM. The direct interactions between $(\sigma^z_l, d_{[l]})$, $(\sigma^z_m, d_{[m]})$, $(\sigma^z_l, d_{[m]})$, and $(\sigma^z_m, d_{[l]})$, are mediated by $h_{[lm1]}$, $h_{[lm2]}$, $h_{[lm3]}$, and $h_{[lm4]}$, respectively, as follows

$$\exp(\sigma^z_l d_{[l]} W''_{l[l]}) = C_{[lm1]} \sum_{h_{[lm1]}} \exp(\sigma^z_l h_{[lm1]} W_{l[lm1]} + h_{[lm1]} d_{[l]} W'_{[lm1][l]}), \tag{91}$$
$$\exp(\sigma^z_m d_{[m]} W''_{m[m]}) = C_{[lm2]} \sum_{h_{[lm2]}} \exp(\sigma^z_m h_{[lm2]} W_{m[lm2]} + h_{[lm2]} d_{[m]} W'_{[lm2][m]}), \tag{92}$$
$$\exp(\sigma^z_l d_{[m]} W''_{l[m]}) = C_{[lm3]} \sum_{h_{[lm3]}} \exp(\sigma^z_l h_{[lm3]} W_{l[lm3]} + h_{[lm3]} d_{[m]} W'_{[lm3][m]}), \tag{93}$$
$$\exp(\sigma^z_m d_{[l]} W''_{m[l]}) = C_{[lm4]} \sum_{h_{[lm4]}} \exp(\sigma^z_m h_{[lm4]} W_{m[lm4]} + h_{[lm4]} d_{[l]} W'_{[lm4][l]}). \tag{94}$$

By applying the gadget Eq. (7), the new W, W' interactions are given by, for small δ_τ (such that $\frac{e^{-J^z_{lm}\delta_\tau}}{\sqrt{\sinh(2J^{xy}_{lm}\delta_\tau)}} > 1$):

$$W_{l[lm1]} = W'_{[lm1][l]} = W_{m[lm2]} = W'_{[lm2][m]} = \tfrac{1}{2}\mathrm{arcosh}\left(\frac{e^{-J^z_{lm}\delta_\tau}}{\sqrt{\sinh(2J^{xy}_{lm}\delta_\tau)}}\right), \tag{95}$$
$$W_{l[lm3]} = -W'_{[lm3][m]} = W_{m[lm4]} = -W'_{[lm4][l]} = \tfrac{1}{2}\mathrm{arcosh}\left(\sqrt{\cosh(2J^{xy}_{lm}\delta_\tau)} \times e^{J^z_{lm}\delta_\tau}\right). \tag{96}$$

How to enforce the constraint $\sigma^z_l + \sigma^z_m = d_{[l]} + d_{[m]}$ ("$i\pi/4, i\pi/8$" trick). Here, we discuss how to design the network to satisfy the constraint $\sigma^z_l + \sigma^z_m = d_{[l]} + d_{[m]}$. We rewrite the sum with the constraint in Eq. (74) as follows (we ignore trivial constant factor):

$$\sum_{\substack{d_{[l]}, d_{[m]} \\ d_{[l]}+d_{[m]}=\sigma^z_l+\sigma^z_m}} \longrightarrow \sum_{d_{[l]}, d_{[m]}} \sum_{h_{[lm5]}, h_{[lm6]}} e^{i\frac{\pi}{4}\left((\sigma^z_l+\sigma^z_m)h_{[lm5]} - h_{[lm5]}(d_{[l]}+d_{[m]})\right)} \times e^{i\frac{\pi}{8}\left((\sigma^z_l+\sigma^z_m)h_{[lm6]} - h_{[lm6]}(d_{[l]}+d_{[m]})\right)}$$
$$= \sum_{d_{[l]}, d_{[m]}} 2\cos\left(\frac{\pi}{4}(\sigma^z_l + \sigma^z_m - d_{[l]} - d_{[m]})\right) \times 2\cos\left(\frac{\pi}{8}(\sigma^z_l + \sigma^z_m - d_{[l]} - d_{[m]})\right) \tag{97}$$

One can easily see that the second line of the equation gives nonzero contribution only when $d_{[l]} + d_{[m]} = \sigma^z_l + \sigma^z_m$.

Summary of the 2d-6h representation. The network changes induced by the bond propagator at each imaginary time step are summarized as follows. Eqs. (87) and (88) imply that $\bar{W}_{lj} = W_{lj} + \Delta W_{lj} = 0$ and $\bar{W}_{mj} = W_{mj} + \Delta W_{mj} = 0$, i.e., all the existing connections between physical spins and hidden neurons vanish. Then, the lth and mth physical spins will be connected to the new hidden neurons $h_{[lm1]}, \ldots, h_{[lm6]}$, The new deep neurons $d_{[l]}$ and $d_{[m]}$ are also connected to $h_{[lm1]}, \ldots, h_{[lm6]}$. In total, we have 16 new connections in the deep Boltzmann network.

By continuing the imaginary time evolution, the neural network grows as in Fig. 2. The number of neurons increases linearly with the number $N_{\rm slice}$ of Suzuki-Trotter time slice. For example, in the case of the one-dimensional Heisenberg model, the total number of deep and hidden neurons are $N_{\rm site}(2N_{\rm slice}+1)$ and $3N_{\rm site}(2N_{\rm slice}+1)$, respectively. The number of

nonzero connections in the network is $8N_{\rm site}(2N_{\rm slice}+1)$. The origin of $2N_{\rm slice}+1$ is coming from the fact that we apply $\mathcal{G}$ propagators $2N_{\rm slice}+1$ times when we apply the second-order Suzuki-Trotter decomposition. The "$i\pi/4, i\pi/8$" trick plays a role to preserve the total magnetization for deep spins at each imaginary-time step, i.e., $\sum_k d_k(t+1) = \sum_k d_k(t)$, where $d(t+1)$ $[d(t)]$ are the deep neurons introduced at $(t+1)$-th $[t$-th$]$ step.

Relationship between the 2d-6h representation and the path-integral quantum Monte Carlo method. In the final part of this section, we discuss the similarity between the 2d-6h representation and the imaginary-time path-integral quantum Monte Carlo method [1]. We will show that, in the 2d-6h representation, the deep neurons can be regard as the additional degrees of freedom along the imaginary time in the path-integral formulation.

In the quantum Monte Carlo simulations using Suzuki-Trotter decomposition [2, 3], the partition function Z is evaluated as

$$\begin{aligned} Z &= \langle \sigma^z(0)|e^{-\beta\mathcal{H}}|\sigma^z(0)\rangle \\ &\simeq \sum_{\sigma^z(0),\dots,\sigma^z(2N_{\rm slice}-1)} \langle \sigma^z(0)|e^{-\mathcal{H}_2\delta_\tau}|\sigma^z(2N_{\rm slice}-1)\rangle\langle \sigma^z(2N_{\rm slice}-1)|e^{-\mathcal{H}_1\delta_\tau}|\sigma^z(2N_{\rm slice}-2)\rangle\dots \\ &\qquad \dots\langle \sigma^z(4)|e^{-\mathcal{H}_2\delta_\tau}|\sigma^z(3)\rangle\langle \sigma(3)|e^{-\mathcal{H}_1\delta_\tau}|\sigma^z(2)\rangle\langle \sigma^z(2)|e^{-\mathcal{H}_2\delta_\tau}|\sigma^z(1)\rangle\langle \sigma^z(1)|e^{-\mathcal{H}_1\delta_\tau}|\sigma^z(0)\rangle \end{aligned} \tag{98}$$

In the evaluation of the matrix element of $\langle \sigma^z(t+1)|e^{-\mathcal{H}_\nu\delta_\tau}|\sigma^z(t)\rangle$ ($\nu = 1$ or 2), in the case of one-dimensional Heisenberg model, it is sufficient to consider one specific bond, $\langle \sigma^z_l(t+1)\sigma^z_m(t+1)|e^{-\mathcal{H}_{lm}\delta_\tau}|\sigma^z_l(t)\sigma^z_m(t)\rangle$. The matrix elements are given by

$$\langle \sigma^z_l(t+1)\sigma^z_m(t+1)|e^{-\mathcal{H}_{lm}\delta_\tau}|\sigma^z_l(t)\sigma^z_m(t)\rangle = e^{J^z_{lm}\delta_\tau}\begin{pmatrix} e^{-2J^z_{lm}\delta_\tau} & 0 & 0 & 0 \\ 0 & \cosh(2J^{xy}_{lm}\delta_\tau) & \sinh(2J^{xy}_{lm}\delta_\tau) & 0 \\ 0 & \sinh(2J^{xy}_{lm}\delta_\tau) & \cosh(2J^{xy}_{lm}\delta_\tau) & 0 \\ 0 & 0 & 0 & e^{-2J^z_{lm}\delta_\tau} \end{pmatrix} \tag{99}$$

in the basis $\{|\uparrow\uparrow\rangle, |\uparrow\downarrow\rangle, |\downarrow\uparrow\rangle, |\downarrow\downarrow\rangle\}$.

On the other hand, the imaginary time evolution in Eq. (2) in the main text [or equivalently, Eq. (16)] can be rewritten as

$$\begin{aligned} \langle \sigma^z|\Psi(\tau)\rangle &= \sum_{\sigma^z(1),\dots,\sigma^z(2N_{\rm slice}+1)} \langle \sigma^z|e^{-\mathcal{H}_1\frac{\delta_\tau}{2}}|\sigma^z(2N_{\rm slice}+1)\rangle\langle \sigma^z(2N_{\rm slice}+1)|e^{-\mathcal{H}_2\delta_\tau}|\sigma^z(2N_{\rm slice})\rangle\dots \\ &\qquad \dots\langle \sigma^z(4)|e^{-\mathcal{H}_1\delta_\tau}|\sigma^z(3)\rangle\langle \sigma^z(3)|e^{-\mathcal{H}_2\delta_\tau}|\sigma^z(2)\rangle\langle \sigma^z(2)|e^{-\mathcal{H}_1\frac{\delta_\tau}{2}}|\sigma^z(1)\rangle\langle \sigma^z(1)|\Psi_0\rangle \end{aligned} \tag{100}$$

by inserting complete basis sets at each time slice. The matrix element used here is exactly the same as that of QMC in Eq. (99). Here, the D dimensional quantum spin system is mapped on the $D+1$ dimensional classical system as in the case of the path integral quantum Monte Carlo method. Because the neuron spins are defined as the classical Ising-type spins, we can represent the summation over $\sigma^z(1),\dots,\sigma^z(2N_{\rm slice}+1)$ by the summation over $N_{\rm site}(2N_{\rm slice}+1)$ neuron spins. Assuming that these $N_{\rm site}(2N_{\rm slice}+1)$ neuron spins are in the deep layer, the imaginary time evolution in Eq. (100) reads

$$\begin{aligned} \langle \sigma^z|\Psi(\tau)\rangle &= \sum_{d(1),\dots,d(2N_{\rm slice}+1)} \langle \sigma^z|e^{-\mathcal{H}_1\frac{\delta_\tau}{2}}|d(2N_{\rm slice}+1)\rangle\langle d(2N_{\rm slice}+1)|e^{-\mathcal{H}_2\delta_\tau}|d(2N_{\rm slice})\rangle\dots \\ &\qquad \dots\langle d(4)|e^{-\mathcal{H}_1\delta_\tau}|d(3)\rangle\langle d(3)|e^{-\mathcal{H}_2\delta_\tau}|d(2)\rangle\langle d(2)|e^{-\mathcal{H}_1\frac{\delta_\tau}{2}}|d(1)\rangle\langle d(1)|\Psi_0\rangle. \end{aligned} \tag{101}$$

The matrix element $\langle d_l(t+1)d_m(t+1)|e^{-\mathcal{H}_{lm}\delta_\tau}|d_l(t)d_m(t)\rangle$ can be reproduced, for example, by the following interaction

$$\begin{aligned} & e^{W''_1(d_l(t+1)d_l(t)+d_m(t+1)d_m(t))}e^{W''_2(d_l(t+1)d_m(t)+d_m(t+1)d_l(t))} \\ & \times\cos\left(\frac{\pi}{4}\left(d_l(t+1)+d_m(t+1)-d_l(t)-d_m(t)\right)\right)\cos\left(\frac{\pi}{8}\left(d_l(t+1)+d_m(t+1)-d_l(t)-d_m(t)\right)\right) \end{aligned} \tag{102}$$

with

$$W''_1 = -\frac{J^z_{lm}\delta_\tau}{2} - \frac{1}{4}\log\sinh(2J^{xy}_{lm}\delta_\tau), \tag{103}$$

$$W''_2 = -\frac{J^z_{lm}\delta_\tau}{2} - \frac{1}{4}\log\cosh(2J^{xy}_{lm}\delta_\tau). \tag{104}$$

This interaction can be mediated by adding hidden neurons and mediating the interactions between $d(t+1)$ and $d(t)$. Then, Eq. (98) can be mapped onto the DBM representation.

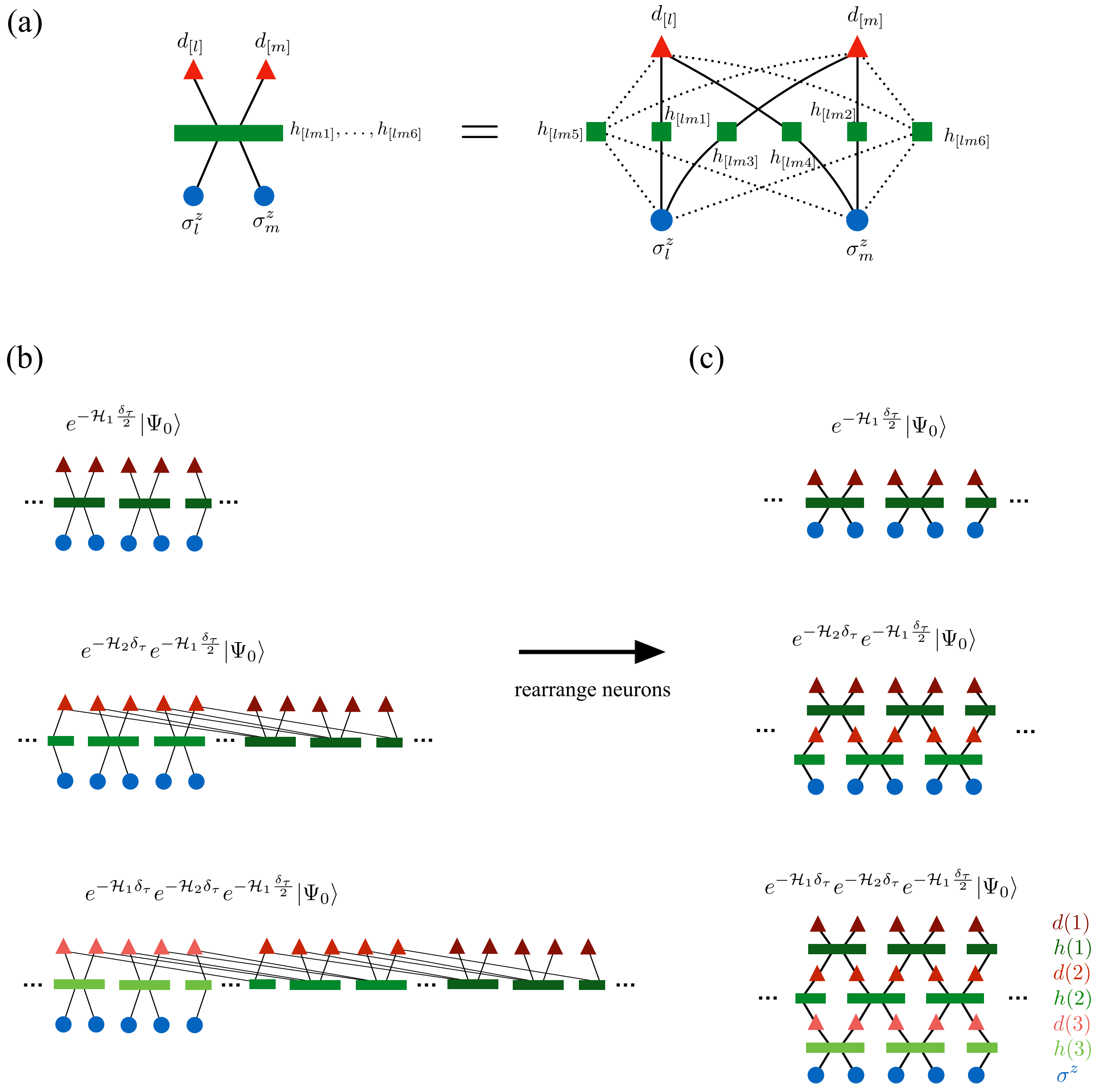

Supplementary Fig. 2. Schematic picture for imaginary time evolution of DBM neural network in the 2d-6h construction. Dots, squares, and triangles indicate physical spins σ_i^z, hidden neurons h_j, and deep neurons d_k, respectively. A set of six hidden neurons are depicted as rectangles. (a) Building block of the imaginary-time evolution. The left part is a simplified picture of the complete figure in the right part. This simplified picture is used in the panels (b) and (c) for the sake of visibility. (b) The imaginary time evolution of the network starting from an empty RBM ($\langle\sigma^z|\Psi_0\rangle$ = const.). The hidden neurons introduced at t-th step ($h(t)$'s) lose their connections to physical spins at $(t+1)$-th step, and instead they get connections to $(t+1)$-th deep neurons ($d(t+1)$'s). (c) When we rearrange the neurons, one can see a clear correspondence between the 2d-6h representation and the path-integral formulation (see the text for detail).

Indeed, the 2d-6h representation presented in this section correspond to this specific DBM construction: In the 2d-6h representation, two deep neurons are introduced for each bond at each imaginary time evolution. Because each imaginary time evolution acts on either even or odd bonds, the number of deep neurons introduced at one step is exactly same as

the number of physical spins. In this case, the deep neurons can be considered as the spin degrees of freedom in the imaginary time layers $d(1),\ldots,d(2N_{\rm slice}+1)$. The interactions in Eqs. (103) and (104) are equivalent to those in Eqs. (89) and (90). The "$i\pi/4, i\pi/8$" trick appears to put constraint to conserve the total magnetization at each layer. Therefore, the 2d-6h representation is equivalent to the path-integral formulation. Indeed, if we rearrange the neurons in this DBM construction (Fig. 2), one can see a clear correspondence between the DBM network and the path-integral formulation. The extended systems including physical spins and deep neurons can be regard as the $D+1$ dimensional classical spin systems mapped from D dimensional quantum systems.

3. 2 deep, 4 hidden *(2d-4h) representation*

Strategy. We first extend DBM in the following way:

$$\Psi_{\bar{\mathscr{W}}}(\sigma^z) = \sum_{\{h,d\}}\sum_{d_{[l]}} P_1(\sigma^z,h)P_2(h,d)e^{\sum_{j,n=l,m}\sigma^z_n h_j\Delta W_{nj}+\sum_j h_j d_{[l]}W'_{j[l]}+\sum_{n=l,m}\sigma^z_n d_{[l]}W''_{n[l]}+\sum_j\sigma^z_l\sigma^z_m h_j d_{[l]}Z_{lmj}}. \tag{105}$$

Here, we have introduced terms which break the standard DBM form, in particular the terms proportional to $W''_{n[l]}$ and Z_{lmj} with $n=l,m$. Those are essential for this construction, and their reduction to the pure DBM will be shown later. Also notice that the sum over j runs through all the hidden neuron sites coupled to σ^z_l and σ^z_m, thus it incorporates nonlocal couplings between hidden variables (h), physical (σ^z) and deep (d) variables. The term proportional to a_i in $P_1(\sigma^z,h)$ is a local site-dependent magnetic-field term in the DBM acting on the physical variables σ^z, which can also flexibly represent any local gauge transformation, if a_i is taken complex. Here we fix a_i to be site-dependent constants, which stay unchanged through the imaginary time evolution. We later use the fact that the gauge transformation $\sigma^x\to-\sigma^x$ and $\sigma^y\to-\sigma^y$ on one of the sublattices (or $J^{xy}\to-J^{xy}$) on a bipartite lattice as in Eq. (41) is equivalent to $a_i=i\pi/2$ if i is on this sublattice and $a_i=0$ on the other sublattice as a special choice of a_i.

In the imaginary time evolution of $\mathscr{H}_{lm}$, we update $\bar{W}_{nj}$ $(n=l,m)$ with the increment ΔW_{nj}, in such a way that $\bar{W}_{nj}=W_{nj}+\Delta W_{nj}$. In addition to the deep variable $d_{[l]}$, we further introduce one additional deep variable $d_{[lm]}$ to recover the standard DBM by transforming the term proportional to W'' and Z, with supplementary four hidden variables.

Derivation for the update of parameters. For $\sigma^z_l\sigma^z_m=-1$, the imaginary time evolution of the bond $\mathscr{H}_{lm}$ is given as

$$\begin{aligned}\langle\sigma^z|e^{-\delta_\tau(J^z_{lm}\sigma^z_l\sigma^z_m+2J^{xy}_{lm}(\sigma^+_l\sigma^-_m+\sigma^-_l\sigma^+_m))}|\Psi_{\mathscr{W}}\rangle &= \Psi_{\mathscr{W}}(\sigma^z)e^{J^z_{lm}\delta_\tau}\cosh(2J^{xy}_{lm}\delta_\tau)\\ &\quad-\Psi_{\mathscr{W}}(\sigma^z_1,\cdots-\sigma^z_l,\cdots-\sigma^z_m\ldots)e^{J^z_{lm}\delta_\tau}\sinh(2J^{xy}_{lm}\delta_\tau) \qquad (106)\\ &= C'\langle\sigma^z|\Psi_{\bar{\mathscr{W}}}\rangle, \qquad (107)\end{aligned}$$

which is equivalent to

$$\sum_{\{h,d\}}\Psi_{\mathscr{W}}\left[1-\tanh(2J^{xy}_{lm}\delta_\tau)e^{-2\sum_{n=l,m}(\sigma^z_n\sum_j h_jW_{nj}+a_n\sigma^z_n)}\right]=C\Psi_{\bar{\mathscr{W}}} \tag{108}$$

and $C=(e^{-J^z_{lm}\delta_\tau}/\cosh(2J^{xy}_{lm}\delta_\tau))C'$. Notice that, here, we keep the bias term a_n in Eq. (1) instead of applying the gauge transformation in Eq. (41).

For $\sigma^z_l\sigma^z_m=1$, we obtain

$$\sum_{\{h,d\}}\Psi_{\mathscr{W}}e^{-2J^z_{lm}\delta_\tau}/\cosh(2J^{xy}_{lm}\delta_\tau)=C\Psi_{\bar{\mathscr{W}}}. \tag{109}$$

To make these imaginary time evolutions exact, W_{nj} $(n=l,m)$ is updated to $\bar{W}_{nj}$ with the increment ΔW_{nj} as $\bar{W}_{nj}=W_{nj}+\Delta W_{nj}$ with

$$\Delta W_{lj}=-\Delta W_{mj}=-\frac{1}{2}(W_{lj}-W_{mj}). \tag{110}$$

The new couplings $W'_{j[l]}$, Z_{lmj} and $W''_{n[l]}$ are also given by

$$W'_{j[l]}=-Z_{lmj}=-\frac{1}{2}(W_{lj}-W_{mj}) \tag{111}$$

and from

$$2(W''_{l[l]} - W''_{m[l]}) = \log[-e^{-2a_{l-m}} \tanh(2J^{xy}_{lm}\delta_\tau)] \tag{112}$$

and

$$2\cosh(W''_{l[l]} + W''_{m[l]}) = \frac{e^{-2J^z_{lm}\delta_\tau - W''_{l[l]} + W''_{m[l]}}}{\cosh(2J^{xy}_{lm}\delta_\tau)}, \tag{113}$$

we obtain

$$W''_{l[l]} = \frac{1}{4}\left[\log\left[-e^{-2a_{l-m}} \tanh(2J^{xy}_{lm}\delta_\tau)\right] + 2\mathrm{arcosh}\left[\frac{e^{-2J^z_{lm}\delta_\tau}}{\sqrt{-2e^{-2a_{l-m}}\sinh(4J^{xy}_{lm}\delta_\tau)}}\right]\right] \tag{114}$$

$$W''_{m[l]} = \frac{1}{4}\left[-\log\left[-e^{-2a_{l-m}} \tanh(2J^{xy}_{lm}\delta_\tau)\right] + 2\mathrm{arcosh}\left[\frac{e^{-2J^z_{lm}\delta_\tau}}{\sqrt{-2e^{-2a_{l-m}}\sinh(4J^{xy}_{lm}\delta_\tau)}}\right]\right] \tag{115}$$

with $a_{l-m} = a_l - a_m$. On a bipartite lattice, to avoid the negative sign (or complex phase) problem we need to keep $W''_{l[l]}$ and $W''_{m[l]}$ real.

This can be achieved by choosing $a_l = 0$ for any l if $J_{lm} < 0$ (ferromagnetic case). For $J_{lm} > 0$ (antiferromagnetic case), $a_l = n\pi i$ with an arbitrary integer n if the site l belongs to the sublattice A and $a_l = (n+1/2)\pi i$ if l belongs to the sublattice B. This local gauge for $J_{lm} > 0$ is equivalent to take $J^{xy}_{lm} \to -J^{xy}_{lm}$ and $a_l = 0$ for any site l as is formulated in Eq.(41). We further note that $W''_{m[l]}$ can be taken positive if we take sufficiently small δ_τ in Eq. (115), with the leading order term $-\log(2J^{xy}_{lm}\delta_\tau)/2$. On the other hand, in Eq. (114), the leading order term is negative ($= -J_{lm}\delta_\tau$).

Recovery of the standard DBM form. To recover the original form of the DBM, we first use Eq. (7) with the replacement $s_1 \to \sigma^z_n$, $s_2 \to d_{[l]}$, $s_3 \to h_{[n]}$, $C \to D_n$, $V \to W''_{n[l]}$ $\tilde{V}_1 \to W_{n[n]}$ and $\tilde{V}_2 \to W'_{[n][l]}$ for $n = l, m$. We have added here two hidden variables $h_{[l]}$ and $h_{[m]}$. Then a solution for D_n, $W_{n[n]}$, and $W'_{[n][l]}$ are represented by using $W''_{n[l]}$ as

$$D_n = \frac{1}{2}\exp[-W''_{n[l]}] \tag{116}$$

$$W_{n[n]} = W'_{[n][l]} = \frac{1}{2}\mathrm{arcosh}(\exp[2W''_{n[l]}]), \tag{117}$$

if $W''_{n[l]}$ is positive (as in the case of $W''_{m[l]}$ for small δ_τ), which gives real $W_{n[n]}$ and $W'_{[n][l]}$. On the other hand, if $W''_{n[l]}$ is negative (as in the case of $W''_{l[l]}$ for small δ_τ), we should take

$$D_n = \frac{1}{2}\exp[W''_{n[l]}] \tag{118}$$

$$W_{n[n]} = -W'_{[n][l]} = \frac{1}{2}\mathrm{arcosh}(\exp[-2W''_{n[l]}]), \tag{119}$$

to give real $W_{n[n]}$ and $W_{[n][l]}$.

To completely recover the original DBM form, we next use Eq. (12) by replacing σ_1 with σ^z_l, σ_2 with σ^z_m, d_1 with $d_{[l]}$, d_2 with $d_{[lm]}$, h_1 with h_j, h_2 with $h_{[lm1]}$, h_3 with $h_{[lm2]}$, and V with Z_{lmj}.

With these solutions, by ignoring the trivial constant factors including D_l and D_m, the evolution is described by introducing two deep and four hidden additional variables $d_{[l]}$, $d_{[lm]}$, $h_{[l]}$, $h_{[m]}$, $h_{[lm1]}$, and $h_{[lm2]}$ as

$$\begin{aligned}\Psi_{\bar{\mathcal{W}}}(\sigma^z) = \sum_{\{\bar{h},\bar{d}\}} P_1(\sigma^z,h)P_2(h,d)\exp\Big[&\sum_{j,n=l,m}\sigma^z_n h_j \Delta W_{nj} + \sum_j h_j d_{[l]} W'_{j[l]} \\ &+ \sum_{n=l,m} h_{[n]}(\sigma^z_n W_{n[n]} + d_{[l]} W'_{[n][l]}) + d_{[lm]}\sum_j h_j Z_{lmj} \\ &+ \frac{i\pi}{4}(h_{[lm1]} + h_{[lm2]})(\sigma^z_l + \sigma^z_m + d_{[l]} + d_{[lm]})\Big],\end{aligned} \tag{120}$$

where $\{\bar{h},\bar{d}\}$ is a set consisting of the existing and new neurons. Equation (120) recovers the standard form of deep Boltzmann machine, where the physical spins σ^z as well as the deep variables d are not interacting each other and couples only to the

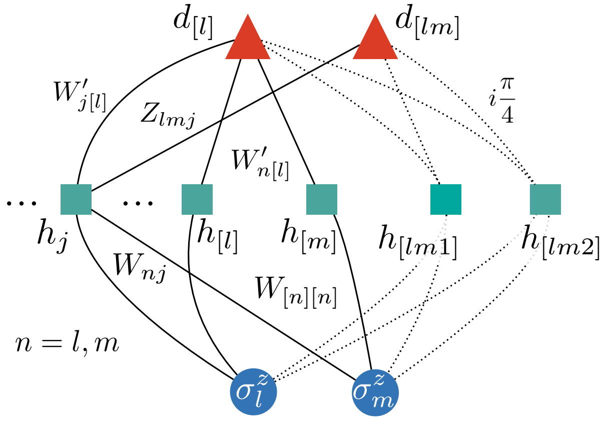

Supplementary Fig. 3. Schematic picture for 2d-4h DBM network. Dots, squares, triangles represent physical (σ_i^z), hidden (h_j), deep (d_k) variables. In 2d-4h construction, both W and W' couplings become nonlocal.

hidden variables h.

Summary. After summing over $\{\bar{h}\}$, we reach

$$\Psi_{\bar{\mathcal{W}}}(\sigma) = \sum_{\{\bar{d}\}} \exp[\sum_{n=l,m} a_n \sigma_n^z] \prod_j [2\cosh[\sum_i \sigma_i^z W_{ij} + \sum_k W'_{jk} d_k + d_{[l]} W'_{j[l]} + d_{[lm]} Z_{lmj}]]$$
$$\times \prod_{n=l,m} \left(2\cosh[\sigma_n^z W_{n[n]} + d_{[l]} W'_{[n][l]}]\right) \left[2\cos[\frac{\pi}{4}(\sigma_l^z + \sigma_m^z + d_{[lm]} + d_{[lm]})]\right]^2 \quad (121)$$

where the parameters W, W' and Z are given in Eqs. (110), (111), (114), (115), and (117) (or (119)).

We have introduced 2 deep and 4 hidden variables. Among them, $h_{[lm1]}$ and $h_{[lm2]}$ are simply to relate $d_{[l]}$ and $d_{[lm]}$ to σ_l^z and σ_m^z. With this trick, one can constrain $d_{[l]} = d_{[lm]}$ for $\sigma_l^z = \sigma_m^z$ and $d_{[l]} = -d_{[lm]}$ for $\sigma_l^z = -\sigma_m^z$. After repeatedly operating Eq. (120), for all the combinations of l, m, the DBM structure becomes nonlocal as we see in Fig. 3. After the sufficiently long imaginary-time evolution, with the analytical sum on $\{h\}$ and the Monte sampling over $\{d\}$, one can obtain the ground state wave function.

Supplementary Note 3: Sampling

Once we have determined specific rules to obtain the parameters of the DBM, the remaining question to be addressed is how to compute expectation values of physical quantities. Consider a quantum operator $\mathcal{O}$, then its expectation value over the DBM is given by the expression

$$\langle \mathcal{O} \rangle = \frac{\sum_{\{\sigma^z,h,h' d,d'\}} \Pi(\sigma^z,h,h',d,d') O_{\mathrm{loc}}(\sigma^z,h,h')}{\sum_{\{\sigma^z,h,h' d,d'\}} \Pi(\sigma^z,h,h',d,d')}, \tag{122}$$

where we have introduced the pseudo-probability density $\Pi(\sigma^z,h,h',d,d') \equiv P_1(\sigma^z,h)P_2(h,d)P_1^\star(\sigma^z,h')P_2^\star(h',d')$, and the "local" estimator $O_{\mathrm{loc}}(\sigma^z,h,h') = \frac{1}{2}\sum_{\{\sigma'^z\}} \langle \sigma^z | \mathcal{O} | \sigma'^z \rangle \left(\frac{P_1(\sigma'^z,h)}{P_1(\sigma^z,h)} + \frac{P_1(\sigma'^z,h')^\star}{P_1(\sigma^z,h')^\star} \right)$. For a large number of spins and hidden/deep units, it is not possible to compute those sums numerically, because of the exponential number of terms involved. However, there are specific cases in which efficient sampling strategies can be devised, allowing to stochastically compute the quantum expectation values. In general, when the DBM weights are all real $\Pi(\sigma^z,h,h',d,d') \geq 0$, and it can be interpreted as an (unnormalized) probability density. Thus, Markov-chain sampling techniques can be applied, similarly to the case of applications in standard machine learning. In the case of complex-valued weights, the straightforward probabilistic interpretation breaks down, and a sign (phase) problem arises. However, there are specific cases in which one can still recover a properly defined probability density, and efficiently sample from it. In the following we describe two main sampling methods based on Markov chain techniques. First, Gibbs sampling, then Metropolis-Hastings sampling. In both cases we discuss when the sign problem can be circumvented.

A. Gibbs sampling

We start discussing a strategy which is the natural generalization of what traditionally used in most applications of DBM in machine learning. The approach is based on Gibbs sampling, a strategy which amounts to generate samples using the exact conditional probabilities for block of variables. In practice, we introduce three kind of moves, which allow to generate a Markov chain of visible, hidden, and deep variables distributed according to $\Pi(\sigma^z,h,h',d,d')$.

1. *Sampling visible spins*

The first kind of move consists in freezing all the hidden and deep variables, and sampling the visible spins σ^z. Specifically, we generate new visible spin configurations according to the conditional probability:

$$\begin{aligned}
\Pi(\sigma^z|h,h',d,d') &= \frac{P_1(\sigma^z,h)P_2(h,d)P_1^\star(\sigma^z,h')P_2^\star(h',d')}{\sum_{\{\tilde\sigma^z\}} P_1(\tilde\sigma^z,h)P_2(h,d)P_1^\star(\tilde\sigma^z,h')P_2^\star(h',d')} \\
&= \frac{P_1(\sigma^z,h)P_1^\star(\sigma^z,h')}{\sum_{\{\tilde\sigma^z\}} P_1(\tilde\sigma^z,h)P_1^\star(\tilde\sigma^z,h')} \\
&= \frac{\Pi_i^N \exp\left\{\sigma_i^z \left[\sum_j \left(h_j W_{ij} + h'_j W^\star_{ij}\right) + 2a_i^{\mathrm{r}}\right]\right\}}{\Pi_i^N 2\cosh\left(\sum_j \left(h_j W_{ij} + h'_j W^\star_{ij}\right) + 2a_i^{\mathrm{r}}\right)}.
\end{aligned}$$

Here, a_i^{r} is a real part of a_i. A particularly appealing aspect of this transition probability is that each visible spin can be treated independently from the others, thus we can update in parallel all visible spins at once. The probability of a given spin to be up for example is:

$$P(\sigma_i^z = 1|h,h',d,d') = \mathrm{Logistic}(2\lambda_i^{[\sigma^z]}), \tag{123}$$

with $\lambda_i^{[\sigma^z]} = \sum_j \left(h_j W_{ij} + h'_j W^\star_{ij}\right) + 2a_i^{\mathrm{r}}$, and $\mathrm{Logistic}(x) = \frac{1}{1+\exp(-x)}$. Thus, during this phase we generate N random numbers η_i uniformly distributed in $[0,1)$, and set the spin $\sigma_i^z = 1$ if $\eta_i < \mathrm{Logistic}(2\lambda_i^{[\sigma^z]})$. For this approach to be feasible, we must have that the $\lambda_i^{[\sigma^z]}$ are real. Necessary conditions for this condition to be satisfied are discussed at the end of this section.

2. Sampling hidden spins

The second type of move consists in freezing visible and deep spins, and sampling hidden variables h and h'. For example, to sample h the transition probability reads:

$$\Pi(h|\sigma^z, h', d, d') = \frac{P_1(\sigma^z, h) P_2(h, d)}{\sum_{\{\tilde{h}\}} P_1(\sigma^z, \tilde{h}) P_2(\tilde{h}, d)}$$
$$= \frac{\Pi_j^M \exp\left[h_j \left(\sum_i \sigma_i^z W_{ij} + b_j + \sum_k d_k W'_{jk}\right)\right]}{\Pi_j^M 2\cosh\left(\sum_i \sigma_i^z W_{ij} + b_j + \sum_k d_k W'_{jk}\right)}.$$

The probability of having $h_j = 1$ is then:

$$P(h_j = 1|\sigma^z, h', d, d') = \text{Logistic}(2\lambda_j^{[h]}), \tag{124}$$

with $\lambda_j^{[h]} = \sum_i \sigma_i^z W_{ij} + b_j + \sum_k d_k W'_{jk}$. Again, one can therefore efficiently update all the M hidden spins at once, without rejection. Analogously, for h' we have $\lambda_j^{[h']} = \sum_i \sigma_i^z W_{ij}^\star + b_j^\star + \sum_k d'_k W'^\star_{jk}$.

3. Sampling deep spins

The final set of moves consists in freezing visible and hidden spins, and sample from deep variables d and d'. For example, to sample d the transition probability is:

$$\Pi(d|\sigma^z, h, h', d') = \frac{P_2(h, d)}{\sum_{\{\tilde{d}\}} P_2(h, \tilde{d})}$$
$$= \frac{\Pi_k^{M'} \exp\left[d_k \left(\sum_j h_j W'_{jk} + c_k\right)\right]}{\Pi_k^{M'} 2\cosh\left(\sum_j h_j W'_{jk} + c_k\right)}.$$

The probability of having $d_k = 1$ is then:

$$P(d_k = 1|\sigma^z, h, h', d') = \text{Logistic}(2\lambda_k^{[d]}), \tag{125}$$

with $\lambda_k^{[d]} = \sum_j h_j W'_{jk} + c_k$. Analogously, we have $\lambda_k^{[d']} = \sum_j h'_j W'^\star_{jk} + c_k^\star$.

4. Overall scheme: alternate block sampling

The overall sampling scheme is therefore realized putting together all those individual Gibbs samplings. In particular, we can devise a two-step block sampling, which takes into account the conditional dependence of all the probabilities previously derived.

The overall sampling scheme then works as follow:

1. Sample h and h', fixing all the other variables. This is realized using the probabilities (124) for all the hidden spins.

2. Sample σ^z,d,d' fixing the values of h and h'. This is realized using the probabilities (123) and (125) for all the visible and deep spins, respectively.

3. Cycle between 1 and 2.

5. Phase problem in the Gibbs scheme

In order to get a consistent sampling scheme, we must have that all the quantities $\lambda_i^{[\sigma^z]}$,$\lambda_j^{[h]}$,$\lambda_j^{[h']}$,$\lambda_k^{[d]}$, $\lambda_k^{[d']}$ are real valued. In the absence of this condition, we have a phase problem, and we cannot directly use a stochastic approach to sample from the DBM. Looking more closely at what conditions are needed, we start noticing that the visible bias can take arbitrary (complex)

values, since only the real parts, a_i^{r}, enter $\lambda_i^{[\sigma^z]}$. In general, there might be specific choices of the DBM parameters which still guarantee absence of phase problem. One possibility is realized, for example, when fixing the total magnetizations in the three layers, i.e. the constraints $\sum_i \sigma_i^z = \sigma_{\mathrm{tot}}^z$, $\sum_j h_j = h_{\mathrm{tot}}$, $\sum_k d_k = d_{\mathrm{tot}}$. We further assume that $\mathrm{Im}(W_{ij}) = W^{\mathrm{I}}$, a constant, as well as $\mathrm{Im}(W'_{jk}) = W'^{\mathrm{I}}$. Then, it is easy to see that the phase problem is avoided when $b_j^{\mathrm{I}} = -\sigma_{\mathrm{tot}}^z W^{\mathrm{I}} - d_{\mathrm{tot}} W'^{\mathrm{I}}$ and $c_k^{\mathrm{I}} = -h_{\mathrm{tot}} W'^{\mathrm{I}}$. Notice that those are just a specific set of conditions, and less stringent ones can be found using other sampling schemes.

When each sample has the imaginary part or negative signs, another possibility of avoiding the phase problem is to take the partial trace summation explicitly so that such partial sum gives always a real nonnegative value. We will discuss this point in more detail in the next section.

B. Metropolis sampling

1. Marginal probability density

Because there are no intralayer interactions in the DBM architecture, one can analytically trace out either one of h, h' and d, d'. Then we get marginal probability density: $\tilde{\Pi}(\sigma^z, h, h') = \sum_{\{d,d'\}} \Pi(\sigma^z, h, h', d, d')$ or $\tilde{\Pi}'(\sigma^z, d, d') = \sum_{\{h,h'\}} \Pi(\sigma^z, h, h', d, d')$. Defining $\tilde{P}(\sigma^z, h)$ and $\tilde{P}'(\sigma^z, d)$ as

$$\tilde{P}(\sigma^z, h) = \sum_{\{d\}} P_1(\sigma^z, h) P_2(h, d) = e^{\sum_i a_i \sigma_i^z + \sum_{ij} \sigma_i^z h_j W_{ij} + \sum_j b_j h_j} \times \prod_k 2\cosh\Big(c_k + \sum_k h_j W'_{jk}\Big) \tag{126}$$

and

$$\tilde{P}'(\sigma^z, d) = \sum_{\{h\}} P_1(\sigma^z, h) P_2(h, d) = \prod_j 2\cosh\Big(b_j + \sum_i \sigma_i^z W_{ij} + \sum_k d_k W'_{jk}\Big) \times e^{\sum_i a_i \sigma_i^z + \sum_k c_k d_k}, \tag{127}$$

respectively, the marginal probability densities are given by

$$\tilde{\Pi}(\sigma^z, h, h') = \sum_{\{d,d'\}} \Pi(\sigma^z, h, h', d, d') = \tilde{P}(\sigma^z, h) \tilde{P}^{\star}(\sigma^z, h'), \tag{128}$$

$$\tilde{\Pi}'(\sigma^z, d, d') = \sum_{\{h,h'\}} \Pi(\sigma^z, h, h', d, d') = \tilde{P}'(\sigma^z, d) \tilde{P}'^{\star}(\sigma^z, d'). \tag{129}$$

With these marginal probability densities, we perform the Metropolis sampling to measure physical quantities. The expectation value of a quantum operator $\mathscr{O}$ is given by

$$\langle \mathscr{O} \rangle = \frac{\sum_{\{\sigma^z,h,h'\}} \tilde{\Pi}(\sigma^z, h, h') \tilde{O}_{\mathrm{loc}}(\sigma^z, h, h')}{\sum_{\{\sigma^z,h,h'\}} \tilde{\Pi}(\sigma^z, h, h')} = \frac{\sum_{\{\sigma^z,d,d'\}} \tilde{\Pi}'(\sigma^z, d, d') \tilde{O}'_{\mathrm{loc}}(\sigma^z, d, d')}{\sum_{\{\sigma^z,d,d'\}} \tilde{\Pi}'(\sigma^z, d, d')} \tag{130}$$

with

$$\tilde{O}_{\mathrm{loc}}(\sigma^z, h, h') = \frac{1}{2} \sum_{\{\sigma'^z\}} \langle \sigma^z | \mathscr{O} | \sigma'^z \rangle \left(\frac{\tilde{P}(\sigma'^z, h)}{\tilde{P}(\sigma^z, h)} + \frac{\tilde{P}(\sigma'^z, h')^{\star}}{\tilde{P}(\sigma^z, h')^{\star}} \right), \tag{131}$$

$$\tilde{O}'_{\mathrm{loc}}(\sigma^z, d, d') = \frac{1}{2} \sum_{\{\sigma'^z\}} \langle \sigma^z | \mathscr{O} | \sigma'^z \rangle \left(\frac{\tilde{P}'(\sigma'^z, d)}{\tilde{P}'(\sigma^z, d)} + \frac{\tilde{P}'(\sigma'^z, d')^{\star}}{\tilde{P}'(\sigma^z, d')^{\star}} \right). \tag{132}$$

2. Phase problem in the Metropolis scheme

An advantage of choosing the marginal probability density is that by taking the summation over h and d, the sign problem can sometimes be avoided even if the DBM has complex parameters. An example is to take the summation over the hidden variables h analytically in the three DBM constructions for the Heisenberg models presented in Sec. 2 B. In all the three cases, only those W and W' couplings used to enforce the constraints are complex-valued, and the summation over h eliminates the negative weight. For example, in the case of the 2d-4h representation in Sec. 2 B 3, though each sample may have a finite imaginary part as in each term of Eq. (120), the total weight becomes real and nonnegative, after the explicit summation over the h degrees of freedom is performed as in Eq. (121).

When the lattice is not bipartite, we can still write down the DBM solutions to exactly follow the imaginary time evolutions. However, in this case, we will have imaginary W and W' parameters even for the units not involved in enforcing the constraints. In this case, the sampling may suffer from sign problem. However, as we discuss in the main text, in contrast to

the conventional quantum Monte Carlo simulations, we can make the number of imaginary time step to reach the ground state short by starting the analytical DBM time evolution [Eq. (16)] from a good stating point $|\Psi_0\rangle$. For example, numerically optimized RBM wave functions can be used for $|\Psi_0\rangle$, or more generally, $|\Psi_0\rangle$ can be wave functions used in the conventional wave function techniques. In this case, before we suffer from a severe sign problems, we might be able to reach the ground state with good statistical accuracy.

3. *Overall scheme*

We sample over σ^z, h, h' [or σ^z, d, d'] with the marginal probability density $\tilde{\Pi}(\sigma^z, h, h')$ [$\tilde{\Pi}'(\sigma^z, d, d')$]. The physical quantities are measured following Eq. (130). In the case of Heisenberg model, after tracing out the h spins, we have constraints over the values of σ^z, d, d'. In that case, a cluster update rather than a local update will be more efficient. In particular, in the 2d-6h representation, since the imaginary-time evolution of the DBM is equivalent to the path-integral formalism, we can apply an efficient cluster update used in the conventional quantum Monte Carlo method, such as so called loop update [4].

Supplementary References

[1] Masuo Suzuki, Seiji Miyashita, and Akira Kuroda, "Monte carlo simulation of quantum spin systems. i," Prog. Theor. Phys. **58**, 1377–1387 (1977).

[2] Masuo Suzuki, "Relationship between d-dimensional quantal spin systems and (d+1)-dimensional ising systems: Equivalence, critical exponents and systematic approximants of the partition function and spin correlations," Prog. Theor. Phys. **56**, 1454 (1976).

[3] H. F. Trotter, "On the product of semi-groups of operators," Proc. Amer. Math. Soc. **10**, 545 (1959).

[4] Hans Gerd Evertz, Gideon Lana, and Mihai Marcu, "Cluster algorithm for vertex models," Phys. Rev. Lett. **70**, 875–879 (1993).
