# Supplementary material for: Constructing exact representations of quantum many-body systems with deep neural networks
Source: Nat Commun. 2018 Dec 14;9:5322. doi: 10.1038/s41467-018-07520-3 (PMC6294148; doi:10.1038/s41467-018-07520-3)
Supplement: Supplementary file 2 — Description of Additional Supplementary Files [file 41467_2018_7520_MOESM2_ESM.pdf]

Description of Additional Supplementary Files

File Name: Supplementary Software 1
Description: tfi.py - Python script to generate a Deep Boltzmann Machine for the Transverse-Field Ising model on a hypercube

File Name: Supplementary Software 2
Description: heis_1d-3h.py - Python script to generate a Deep Boltzmann Machine for the antiferromagnetic Heisenberg model in one dimension, following the 1d-3h construction as described in the paper

File Name: Supplementary Software 3
Description: heis_2d-4h.py - Python script to generate a Deep Boltzmann Machine for the antiferromagnetic Heisenberg model in one dimension, following the 2d-4h construction as described in the paper

File Name: Supplementary Software 4
Description: heis_2d-6h.py - Python script to generate a Deep Boltzmann Machine for the antiferromagnetic Heisenberg
